# Depth-dependent soil water-salt relationships in the Hetao Irrigation District, arid Northwest China: Implications for sustainable management

**DOI:** 10.1371/journal.pone.0360976

**Published:** 2026-10-20

**Authors:** Yuanqing Cao, Huihui Wang, Xuan Chen, Li Zhu

**Affiliations:** 1 Department of Mathematics and Computer Science, Hetao College, Bayannur, Inner Mongolia, China; 2 Department of Hydraulic and Civil Engineering, Hetao College, Bayannur, Inner Mongolia, China; PLOS: Public Library of Science, UNITED STATES OF AMERICA

## Abstract

Soil salinization threatens agricultural productivity in arid gravity irrigation systems worldwide, yet the depth-stratified relationships between soil water and salt content remain poorly characterized. We monitored soil moisture and electrical conductivity (EC) at six depths (0–100 cm) across 15 sites in the Hetao Irrigation District, China, during the 2025 growing season. Our results indicate a depth-dependent divergence in water–salt associations. The EC-derived salinity index varied from 0.08 to 42.91 g/kg, exhibiting extreme spatial heterogeneity (coefficient of variation (CV) = 185.2%) across the salinity gradient. However, moisture-salinity relationships showed marked depth dependency. Surface soils (0–20 cm) exhibited non-statistically significant correlations (*r* = 0.07), whereas deep layers (60–100 cm) showed a statistically significant but weak positive correlation (*r* = 0.25, *p* < 0.01, *R*^2^ = 0.06), indicating limited explanatory power. This pattern is consistent with a subtle tendency of downward salt redistribution. The EC-derived salinity index showed a 42% increase in deep layers (from 1.59 to 2.26 g/kg) over the season, compared to a 136% fluctuation in surface layers. These results highlight limitations of surface-focused monitoring approaches and indicate patterns consistent with subsurface salt redistribution. Compared with previous studies, this work provides a depth-resolved, multi-site observational dataset combined with a stratified analytical approach, offering additional evidence for interpreting soil salinity dynamics under heterogeneous field conditions.

## Introduction

Soil salinization affects ~20% of irrigated lands worldwide, expanding at 1–2 million hectares annually [1–6]. In China, 36 million hectares are salt-affected [7], with the Hetao Irrigation District—one of China’s three largest irrigation districts—exemplifying this crisis: over 60% of its area suffers irrigation-induced salinity [8,9], threatening long-term productivity.

Understanding soil water-salt relationships is fundamental to managing salinization, as water movement governs salt transport through upward capillary flow (evaporation-driven) and downward leaching (irrigation/precipitation-induced) [10,11]. These fluxes create complex spatial and temporal salt distribution patterns varying with depth, texture, groundwater, and management [12]. However, most studies focus on surface soil (0–20 cm) or assume uniform profiles, missing critical deeper-layer dynamics where salts accumulate during leaching and redistribute later [13]. Deep-layer salt accumulation has been reported to potentially influence long-term sustainability in previous studies: subsurface layers may approach storage limits under sustained irrigation, reducing leaching effectiveness and increasing upward redistribution to roots [4,5]. This may increase irrigation demand and exacerbate salinity management challenges [14].

Electrical conductivity (EC) offers rapid salinity assessment [15,16], though moisture effects and depth-dependent ionic variations require careful calibration [17–20].

Seasonal dynamics add complexity to salinity management. In gravity-fed systems like Hetao, managed flooding (May-July) leaches salts [21], but effectiveness varies with climate and depth. Traditional approaches assume sufficient leaching fraction will maintain acceptable salinity levels by flushing salts downward [22]. However, irrigation may redistribute salts deeper rather than removing them [23], and vadose zone salt storage constrains long-term leaching effectiveness [24,25]. This raises fundamental questions about long-term sustainability and the ultimate fate of leached salts. This concerns regions like Hetao, where shallow groundwater tables (1.5–3.5 m) may intercept downward salts and create reservoirs that return to the profile through capillary rise.

While subsurface salt accumulation and depth-dependent salinity dynamics have been reported in previous studies, existing research is often based on limited sampling depth, single-site observations, or simplified assumptions of soil profile uniformity.

This study differs from previous work in three main aspects. First, it is based on a multi-site field dataset (15 locations) covering the full soil profile (0–100 cm) across a wide salinity gradient. Second, it applies a depth-stratified analytical framework that combines correlation analysis with moisture-class group comparison to better capture patterns under heterogeneous field conditions. Third, the study adopts a pattern-oriented interpretation, focusing on identifying consistent signals in observational data rather than establishing strong predictive or mechanistic relationships.

These aspects allow a more comprehensive examination of depth-dependent water–salt relationships and provide additional evidence for understanding salinity redistribution processes in arid irrigation systems.

## Materials and methods

### Study area

The Hetao Irrigation District (40°13′–41°16′N, 106°20′–109°19′E) occupies 574,000 ha in Inner Mongolia, northwestern China, between the Yellow River (south) and Yinshan Mountains (north). The region has a temperate continental monsoon climate: cold winters and hot, dry summers. Mean annual temperature is 6.8℃ (January: −11.6℃; July: 23.2℃). Annual precipitation averages 139 mm (range: 130–215 mm), concentrated July–September, while potential evapotranspiration reaches 2,200 mm, creating severe moisture deficits requiring intensive irrigation.

The irrigation system diverts 4–5 billion m^3^ annually from the Yellow River through over 10,000 km of canals. Irrigation (April–September) typically applies 450–600 mm across 8–12 events depending on crop requirements and soil conditions. The drainage system comprises 15 primary channels (807 km total) plus secondary/tertiary networks, but capacity remains insufficient in many areas, contributing to shallow groundwater and salinization [26].

Soils are predominantly alluvial deposits from Yellow River sediments, classified as Aridisols and Entisols (USDA taxonomy). Textures range from sandy loam to silt loam, with low organic matter (8–15 g/kg) typical of arid soils. Bulk density averages 1.35–1.45 g/cm^3^; hydraulic conductivity varies 0.5–5 cm/h by texture. Groundwater depth varies 1.5–3.5 m, fluctuating 0.5–1.0 m seasonally with irrigation and drainage.

The dominant cropping pattern is spring wheat (April–July) followed by sunflower (July–October), though maize and other crops occur locally. This double-cropping system maximizes productivity but intensifies water demand and complicates salinity management by requiring irrigation when evaporative demand peaks [27].

### Sampling design

We established 15 monitoring sites (spring 2025) capturing spatial salinity heterogeneity across representative farmland. Sites were selected via stratified sampling based on preliminary surveys with a portable salinity sensor (TZS-ECW, Zhejiang Top Cloud-agri Technology, China). This process deliberately included locations from non-saline to highly saline, preventing low-salinity bias. This design targeted gradient analysis rather than regional averages. Field sampling at all 15 monitoring sites was conducted with the institutional support and permission of the local agricultural extension station (Hetao Irrigation District Administration). No specific permits were required for the described field studies as the sampling locations were situated on cooperative experimental farmlands.

All sites had wheat-sunflower rotation with similar management. Sites spaced 0.5–8 km across ~150 km^2^. Based on these preliminary survey values, the 15 monitoring sites were initially categorized into four salinity classes to maximize gradient coverage: non-saline (EC < 400 µS/cm, 7 sites), slightly saline (EC 400–1000 µS/cm, 3 sites), moderately saline (EC 1000–2000 µS/cm, 2 sites), and highly saline (EC > 2000 µS/cm, 3 sites).

Sampling occurred monthly April 15–September 15, 2025 (excluding May). For consistency, samples were collected days 15–20 of each month, ≥ 5 days after irrigation or rainfall. This timing minimizes transient moisture effects while reflecting typical growth-period conditions. May was excluded due to continuous irrigation preventing site access. The dataset comprises April, June, July, August, and September measurements.

At each site, we sampled six depths: 0–10, 10–20, 20–40, 40–60, 60–80, and 80–100 cm. These intervals capture near-surface dynamics (0–20 cm), active rooting zone (20–60 cm), and deeper salt sinks (60–100 cm). Three replicate cores within a 2-m radius were extracted using a stainless-steel auger (Type TY-1, Nanjing Soil Instrument Factory, China; 3 cm diameter, 100 cm length) and composited by depth. Each depth yielded 500–800 g soil. Samples were sealed in labeled bags and transported within 6 hours. This yielded 450 samples (15 sites × 6 depths × 5 months). Permanent markers ensured consistent sampling locations.

### Laboratory analyses

In the laboratory, samples were divided into subsamples. One subsample determined moisture: field-moist samples were weighed, oven-dried (105℃, 24 h to constant weight), and re-weighed for gravimetric water content. Remaining soil was air-dried (7–10 days), ground through a 2-mm sieve, and stored for analysis.

Electrical conductivity (EC_1:5_) was measured in 1:5 soil: water extracts following standard protocols [28]. Air-dried soil (20 g) was mixed with deionized water (100 mL), shaken (5 min), settled (30 min), and filtered (quantitative filter paper, slow speed, Great Wall, China). EC was measured at 25℃ (DDS-307A meter, Rex Electric Chemical, Shanghai; ± 1% accuracy).

An EC-derived salinity index (hereafter referred to as derived salinity) was estimated from EC_1:5_ measurements using a published conversion equation calibrated for Hetao Irrigation District soils [29]:


$$\begin{array}{c} Derived\;Salinity\;(g/kg)=0.0037657\times {EC}_{1:5}\;(\mu S/cm)-0.2405 \end{array}$$ (1)

Where Derived Salinity is the EC-derived salinity (g/kg) and EC_1:5_ is the electrical conductivity (µS/cm). This relationship derives from regional linear regression between EC_1:5_ and gravimetric salts, and is widely adopted for rapid, non-destructive estimation [16,28–30]. Our objective is to examine how soil moisture dynamics associate with the spatial heterogeneity and profile redistribution of these indices, recognizing that weak correlations (e.g., R^2^ = 0.06) reflect the complex, multifactorial nature of salinity dynamics in arid systems.

Methodological limitations of the EC-based approach. Because no direct gravimetric quantification or ion-specific analyses were conducted, three constraints apply to this methodology. First, the estimated values represent a relative index of salinity rather than precise in situ salt mass, carrying inherent regional conversion uncertainty. Second, the spatial and temporal variability of specific ionic compositions (e.g., SO_4_^2−^ , HCO_3^−^_) cannot be resolved from EC alone. Third, sodicity-related hazards (e.g., sodium adsorption ratio, exchangeable sodium percentage) cannot be assessed. These limitations must be considered when interpreting quantitative results and management implications.

### Data analysis

Descriptive statistics (mean, standard deviation (SD), CV, range) were calculated for moisture, EC, and the derived salinity index (n = 450), stratified by depth or month. Data distributions were examined using histograms and quantile–quantile plots. Although EC and salinity were right-skewed, Pearson correlation was appropriate given sample size and robustness to moderate non-normality.

Pearson correlations quantified relationships among moisture, EC, and the derived salinity index for the full dataset and three depth classes: surface (0–20 cm), transition (20–60 cm), subsurface (60–100 cm). Spearman rank correlations confirmed robustness to non-normality. Significance was evaluated at *α* = 0.05; effect sizes followed Cohen’s guidelines [31] (small: *r* ≤ 0.10; medium: *r* ≤ 0.30; large: *r* > 0.50).

Multiple linear regression and correlation analyses were used to examine how independent explanatory variables (moisture and depth) relate to the EC-derived salinity index. Model performance used *R*^2^ and root mean square error (RMSE). Assumptions were assessed via residual diagnostics; stability via leave-one-out cross-validation.

Temporal variations used time-series visualizations and analysis of variance (ANOVA), with site as a blocking factor for repeated measurements. Pairwise monthly differences used Tukey’s Honestly Significant Difference (HSD) test.

Compared with conventional regression-based approaches commonly used in salinity studies, this study adopts a group-comparison analytical framework to examine how EC distributions vary across moisture classes within each depth category. To overcome the limitation of weak linear correlation (*r* = 0.25, *R*^2^ = 0.06) and to avoid circular regression, a group-comparison strategy was adopted as recommended for pattern recognition in heterogeneous field data. Soil samples were stratified by (1) depth intervals (0–20 cm: surface; 20–60 cm: transition; 60–100 cm: deep), and (2) moisture classes defined by empirical terciles of the dataset within each depth category (Low: ≤ 33rd percentile; Medium: 33rd–67th percentile; High: > 67th percentile).

Differences in EC among moisture classes within each depth were evaluated using one-way ANOVA with Bonferroni-corrected pairwise t-tests (*α* = 0.05/3 = 0.0167). This approach shifts the analytical focus from regression prediction to response pattern analysis—examining how EC signals vary across moisture gradients rather than attempting to predict absolute salt mass from moisture.

Box plots were generated to visualize EC distributions across moisture classes for each depth category. This group-comparison approach allows identification of consistent EC-moisture associations even when overall linear correlations are weak, as is expected in complex, multifactorial arid soil systems where salinity dynamics are influenced by numerous interacting processes (irrigation timing, evaporation, capillary rise, soil texture heterogeneity). This approach emphasizes pattern detection rather than predictive modeling, which is particularly suitable for heterogeneous field datasets where linear relationships are often weak.

All analyses used Python 3.13.11 (NumPy, Pandas, SciPy, statsmodels) [32]. To address the inherent circularity, we emphasize that our statistical models do not treat EC and derived salinity as independent variables. Rather, regressions evaluate how environmental factors interact with the EC signal. Consequently, all results are interpreted as spatial patterns of soluble ions rather than predictive mechanistic models.

## Results

### Spatial variability and general characteristics

All salinity values reported in this section are EC-derived estimates (Eq. 1) and are intended to characterize variability across the sampled salinity gradient rather than represent regional averages.

Soil properties exhibited substantial spatial heterogeneity (Table 1). Gravimetric moisture averaged 0.157 g/g but varied widely (range 0.005–0.442, CV = 49%), reflecting soil texture, irrigation, weather, and groundwater depth. The extremely low minimum value (0.005 g/g) represents near-air-dry conditions in surface soils during hot, dry periods between irrigations, while the maximum (0.442 g/g) occurred in fine-textured soils shortly after irrigation. Electrical conductivity showed extreme variability (CV = 167.4%), ranging nearly three orders of magnitude from 85 to 11,460 µS/cm. Similarly, the derived salinity index spanned from 0.08 to 42.91 g/kg (mean = 2.26 g/kg, CV = 185.2%), indicating the presence of both non-saline and severely salt-affected locations within the study area.

**Table 1:** Summary statistics for soil properties across all sampling sites, depths, and months (n = 450).

Property	Mean	SD	Min	Max	CV (%)
**Soil Moisture (g/g)**	0.16	0.08	0.00	0.44	49.0
**EC (μS/cm)**	663.81	1111.39	85.00	11460.00	167.4
**EC-derived Salinity (g/kg)**	2.26	4.19	0.08	42.91	185.2

The EC-derived salinity index is used as the primary salinity variable in all analyses. EC_1:5_ values are reported only where direct comparison with existing literature requires conductivity units. Because these two metrics are linear transformations of the same measurement, they are not used simultaneously as independent predictors in statistical models. The spatial distribution of soil salinity across the 15 sites revealed distinct potential zones of EC-derived salinity redistribution that appear to be controlled by topography, drainage infrastructure, and groundwater conditions. Three sites (YG6, YG9, YG13) consistently exhibited high EC values (>1000 µS/cm on average), suggesting chronic salinization problems potentially linked to inadequate drainage, proximity to saline groundwater, or accumulation in topographic depressions. These sites showed the highest spatial variability, with coefficients of variation exceeding 200%. Conversely, seven sites (YG1, YG2, YG3, YG8, YG10, YG14, YG15) maintained relatively low salinity (EC < 400 µS/cm) throughout the season, demonstrating effective natural or managed drainage. The remaining sites showed intermediate or variable conditions, with salinity levels fluctuating seasonally in response to irrigation and evapotranspiration cycles.

### Depth-stratified moisture-salinity relationships

The dataset exhibited a highly skewed salinity distribution, with values ranging from 0.08 to 42.91 g/kg, reflecting the extreme spatial heterogeneity typical of arid irrigation districts. Depth-stratified analysis reveals distinct moisture–salinity association patterns across the soil profile. Surface layers (0–20 cm) exhibited weak and non-statistically significant correlations between soil moisture and the derived salinity index (*r* = 0.07, *P* > 0.05), indicating that surface salinity dynamics are driven principally by rapid evaporation and irrigation pulses rather than consistent moisture-driven processes potentially associated with leaching, although this cannot be directly verified. In contrast, the relationship shifted significantly with depth. The transition zone (20–60 cm) showed intermediate behavior (*r* = 0.12, *P* > 0.05), reflecting a dynamic equilibrium. Critically, the deep subsurface layers (60–100 cm) showed a statistically significant but weak positive correlation (r = 0.25, P < 0.01). However, the low coefficient of determination (R^2^ = 0.06) indicates limited practical significance, with soil moisture explaining only a small fraction of the variability in deep-layer salinity. This suggests that while a statistical association exists, moisture alone is insufficient to characterize salinity dynamics in these layers. These depth-stratified patterns are visually consistent with the moisture–EC relationships illustrated in Fig 1.

**Fig 1:**
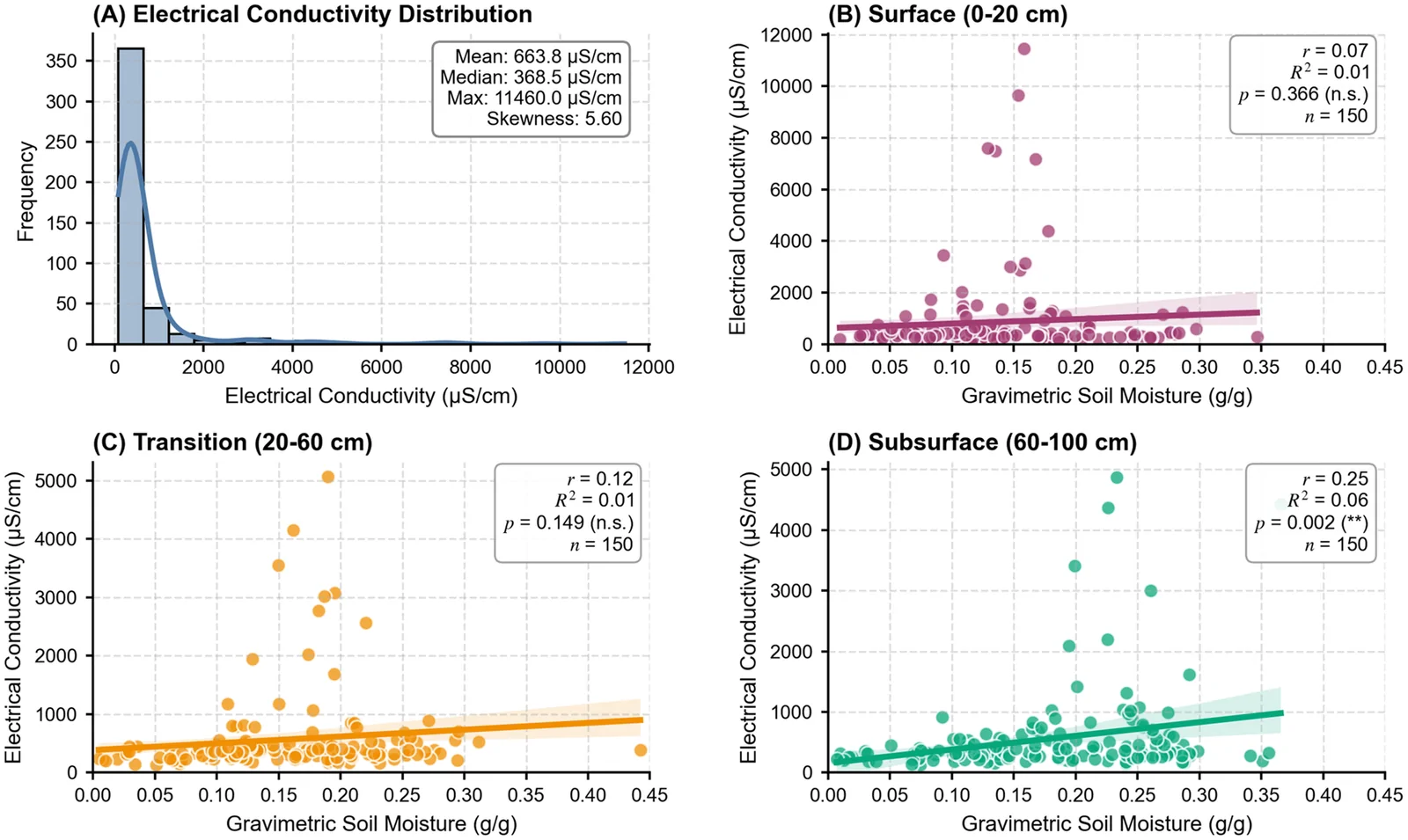
Statistical characteristics and depth-dependent moisture–EC associations across the soil profile. (A) Frequency distribution of electrical conductivity (EC) across all layers (n = 450). (B–D) Linear relationships between gravimetric soil moisture and EC for the (B) Surface (0–20 cm), (C) Transition (20–60 cm), and (D) Subsurface (60–100 cm) layers. Shaded areas represent 95% confidence intervals.

The moisture-class stratification results are presented in Fig 2 and provide additional insight into EC patterns across the soil profile. One-way ANOVA revealed statistically significant differences in EC among moisture classes for all three depth categories (Surface: *F* = 3.42, *p* = 0.035; Transition: *F* = 3.94, *p* = 0.022; Subsurface: *F* = 4.38, *p* = 0.014; Fig 2). In the deep layers (60–100 cm), pairwise comparisons showed that both Medium and High moisture classes had significantly higher EC values than the Low moisture class (*p* < 0.01 after Bonferroni correction), with mean differences of 411 µS/cm and 291 µS/cm, respectively. This group-level distinction indicates that EC signals differ across moisture classes at all depths. However, because the derived salinity index is a linear transformation of EC, these group comparisons should be interpreted as patterns in the ionic signal rather than evidence of independently confirmed moisture-driven salt redistribution.

**Fig 2:**
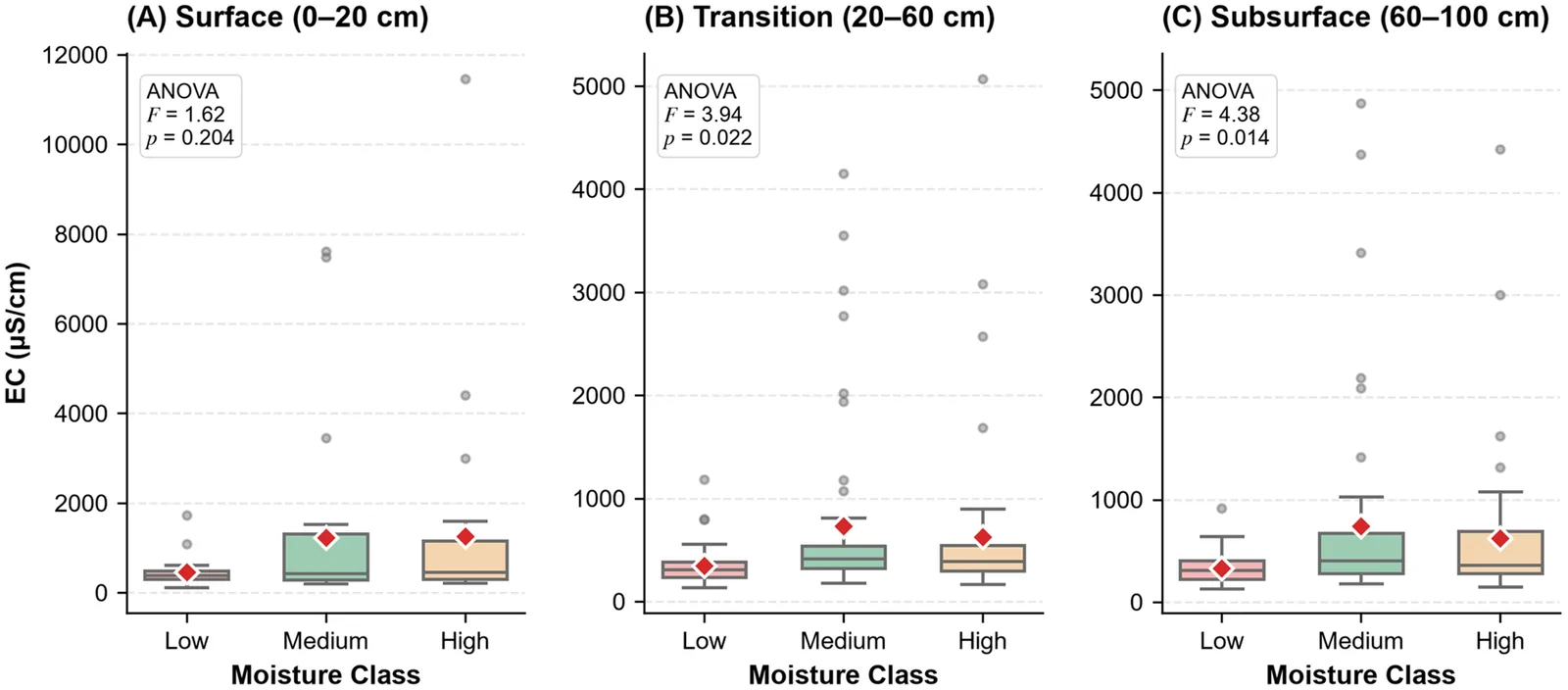
EC distribution across moisture classes by depth category. (A) Surface layer (0–20 cm), (B) transition layer (20–60 cm), and (C) subsurface layer (60–100 cm). Box plots display the median (horizontal line), interquartile range (box), 1.5 × IQR (whiskers), and outliers (gray points); red diamonds indicate group means. Moisture classes (Low, Medium, High) represent empirical terciles within each depth interval.

Average soil properties varied systematically with depth (Table 2). Moisture content increased progressively from 0.129 g/g in surface soils to 0.190 g/g at 80–100 cm depth, consistent with typical evaporative gradients in arid-zone profiles.

**Table 2:** Mean soil properties by depth interval averaged across all sites and sampling months.

Depth (cm)	Moisture (g/g)	EC (μS/cm)	EC-derived Salinity (g/kg)
**0-10**	0.129 ± 0.071	980.3 ± 1810.5	3.45 ± 6.82
**10-20**	0.135 ± 0.068	731.2 ± 1396.5	2.51 ± 5.26
**20-40**	0.145 ± 0.065	598.7 ± 847.2	2.01 ± 3.19
**40-60**	0.170 ± 0.079	538.8 ± 574.6	1.79 ± 2.16
**60-80**	0.175 ± 0.076	546.7 ± 705.4	1.82 ± 2.66
**80-100**	0.190 ± 0.084	587.1 ± 758.2	1.97 ± 2.86

The EC-derived salinity index showed more complex depth-dependent patterns (with corresponding EC_1:5_ values reflecting the same signal), reaching its maximum in surface layers (0–10 cm: 3.45 g/kg; EC = 980 µS/cm), declining to a minimum around 20–40 cm depth (2.01 g/kg; EC = 599 µS/cm), and increasing again in the deeper layers (80–100 cm: 1.97 g/kg; EC = 587 µS/cm). This bimodal salinity distribution—with peaks at both surface and depth—indicates dual accumulation mechanisms. Surface enrichment results from evaporative concentration, well-documented in arid agriculture. The deeper accumulation suggests that current irrigation practices may be associated with subsurface salt loading.

The depth of minimum salinity (20–40 cm) may represent a notable zone where irrigation may facilitate leaching-related processes, although this cannot be directly verified, located deep enough to escape intense evaporative enrichment but shallow enough to receive regular leaching from irrigation water percolating downward.

### Seasonal dynamics across depth zones

Having established the depth-dependent moisture-salinity relationships, we now examine how these patterns evolve temporally over the growing season. Salt redistribution processes operate at different timescales across soil depths. Because monthly sampling was conducted without observations in May — the period most likely corresponding to peak leaching activity — the dataset captures net seasonal changes rather than the redistribution dynamics themselves. Consequently, conclusions about leaching effectiveness, peak salt movement, and irrigation-driven redistribution pathways are not directly supported by the data and should be treated as inferential.

The spatiotemporal distribution of soil salinity across the growing season is visualized in Fig 3, which provides a comprehensive heatmap showing the temporal evolution of the derived salinity index at different depths from April to September. This visualization reveals distinct accumulation patterns in three functional zones of the soil profile.

**Fig 3:**
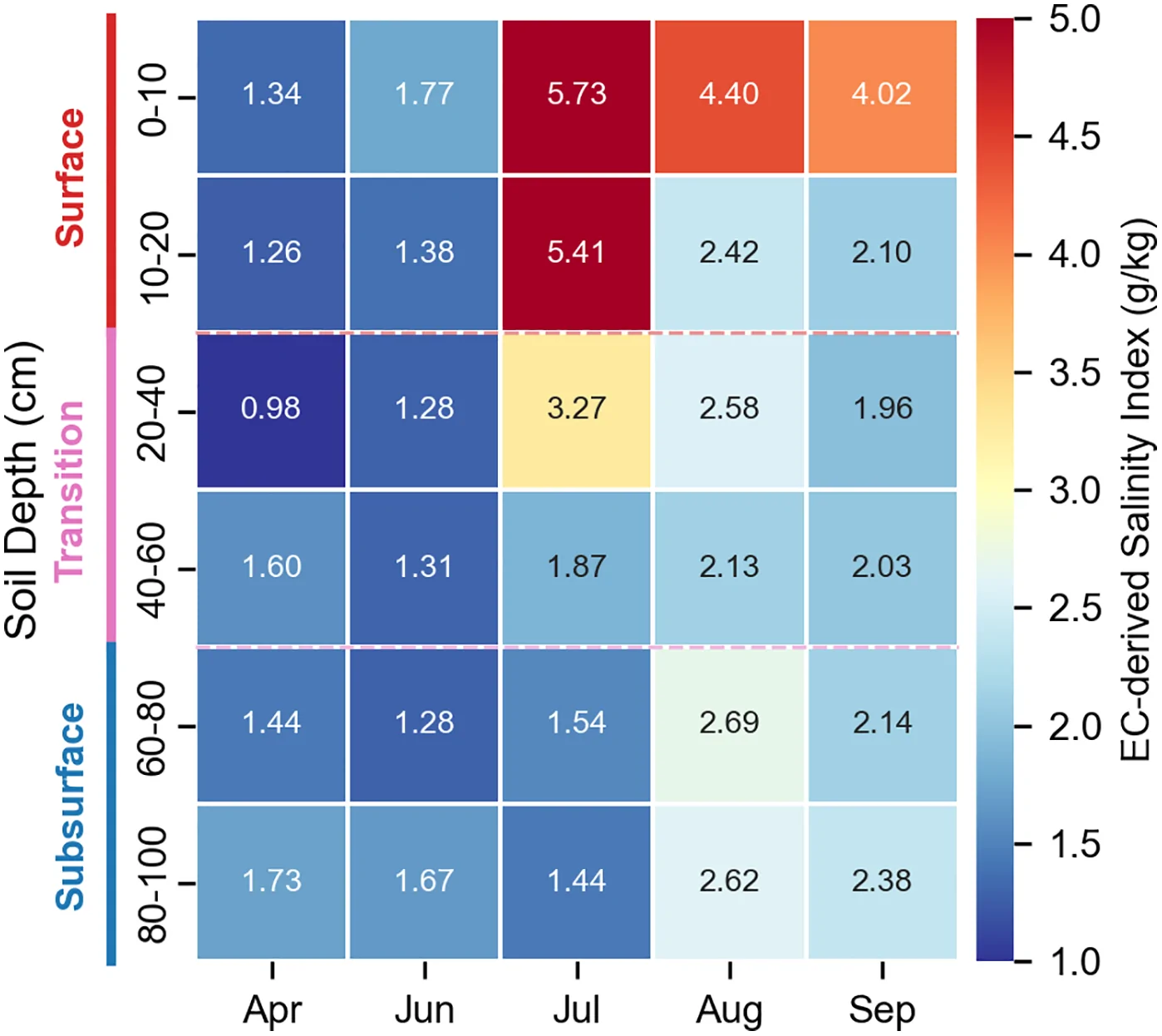
Seasonal heatmap of depth-resolved salinity dynamics (April–September 2025).

The spatiotemporal distribution pattern illustrates these depth-dependent dynamics across the growing season.

Seasonal patterns revealed substantial temporal variability (Fig 4, Table 3). Moisture content peaked in June (0.192 g/g) during the main irrigation period, declined through July–August (0.143–0.160 g/g) as crop water uptake and evapotranspiration exceeded inputs, and reached annual lows in September (0.136 g/g).

**Table 3:** Monthly averages of soil properties across all sites and depths.

Month	Moisture (g/g)	EC (μS/cm)	EC-derived Salinity (g/kg)
**April**	0.156 ± 0.083	433.1 ± 435.0	1.39 ± 1.64
**June**	0.192 ± 0.082	448.8 ± 263.7	1.45 ± 0.99
**July**	0.143 ± 0.054	916.7 ± 1833.5	3.21 ± 6.90
**August**	0.160 ± 0.084	808.8 ± 1145.2	2.81 ± 4.31
**September**	0.136 ± 0.066	711.6 ± 1053.9	2.44 ± 3.97

**Fig 4:**
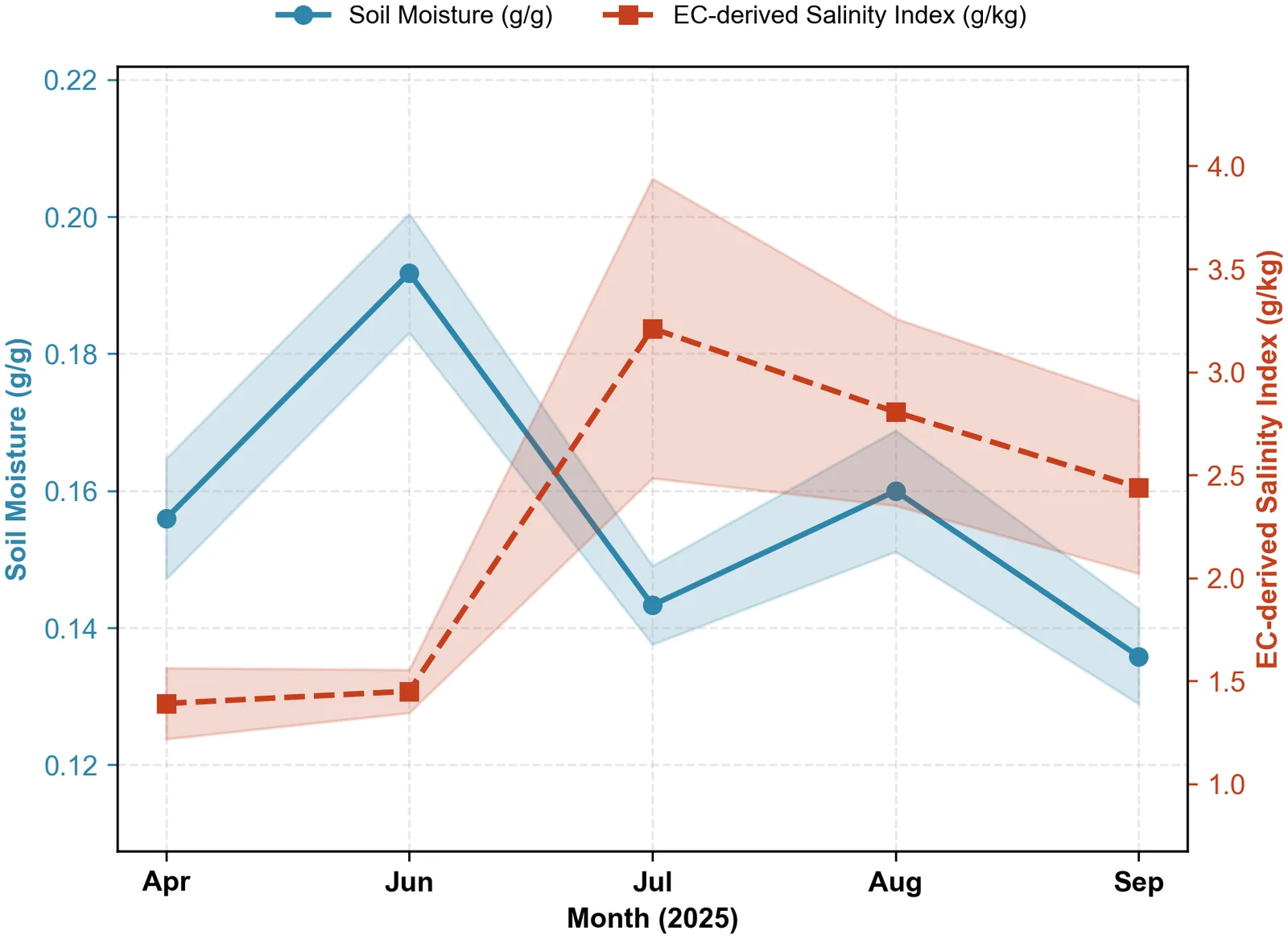
Seasonal dynamics of soil properties. Monthly changes in mean soil moisture (blue) and the EC-derived salinity index (red) averaged across all sites (April–September 2025). Shaded regions represent the standard error of the mean.

Salinity showed inverse trends. The lowest values occurred in April (1.39 g/kg) following winter snowmelt. The EC-derived salinity index increased during June (1.45 g/kg) despite heavy irrigation, then peaked in July (3.21 g/kg) when hot, dry conditions drove intense evapotranspiration. A modest decline in August–September (2.81–2.44 g/kg) corresponded with reduced evaporative demand.

Surface soils (0–20 cm) showed dramatic variability: salinity increased 21% from April to June despite irrigation, then surged 329% by July, reflecting evaporative dominance during peak summer heat.

Subsurface layers (60–100 cm) exhibited complex seasonal patterns: salinity decreased slightly from April (1.59 g/kg) to June (1.47 g/kg), remained relatively stable through July (1.47 g/kg), then increased dramatically in August (2.65 g/kg) due to late-season redistribution of EC-derived salinity, before declining moderately to September (2.26 g/kg). The seasonal increase of 42% in deep layers from April to September indicates a net seasonal redistribution of EC-derived salinity within the soil profile. This subsurface accumulation may indicate early subsurface salt storage, with salts at 60–100 cm depth potentially available for upward transport during dry periods, although its longer-term implications require multi-season validation.

To provide detailed quantitative analysis of these seasonal patterns, Fig 5 presents vertical profiles and temporal evolution curves for both moisture and the derived salinity index across the three depth zones. This detailed view reveals the specific timing and magnitude of EC signal redistribution events, showing three distinct zonal behaviors across the soil profile.

**Fig 5:**
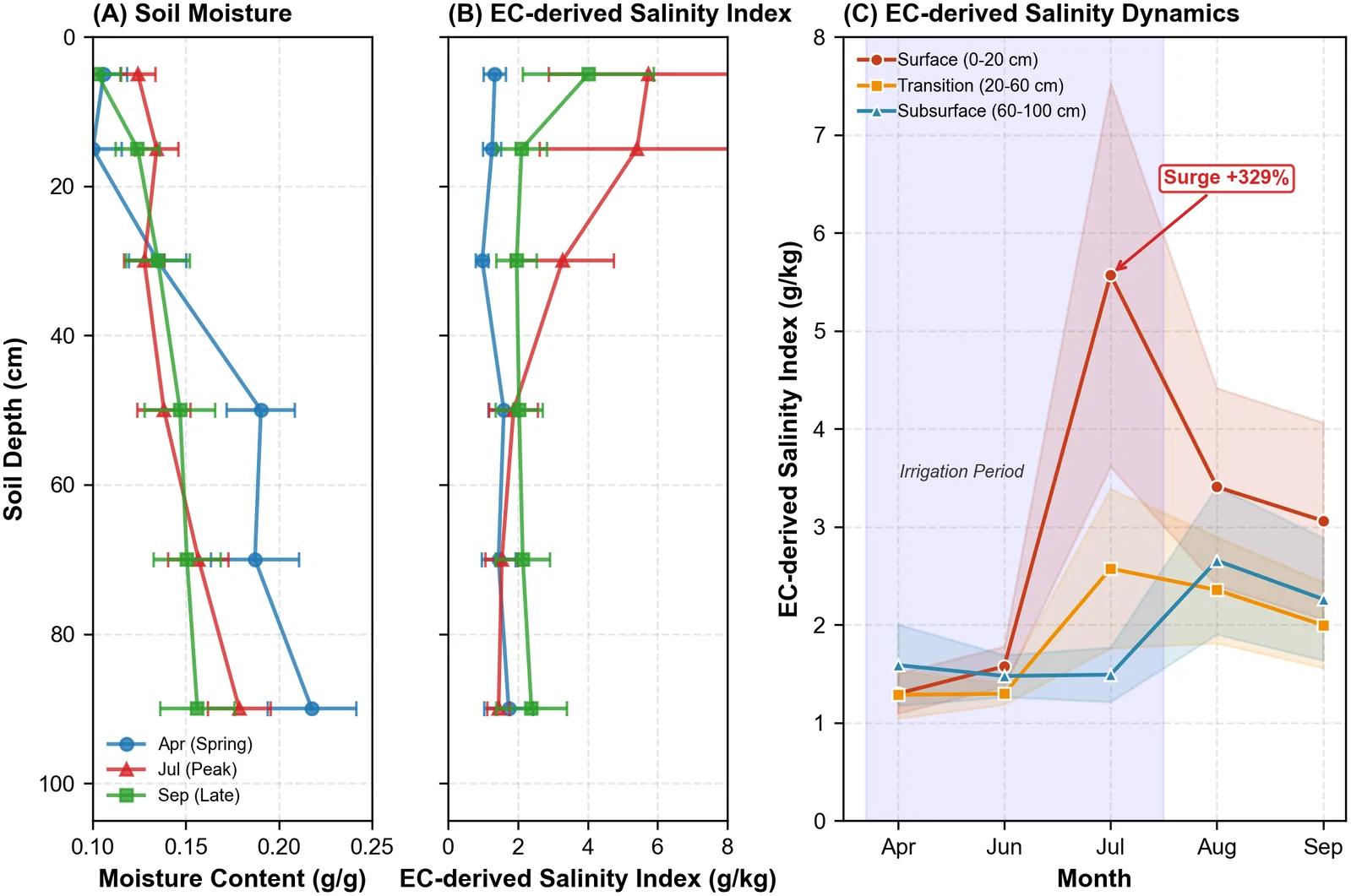
Seasonal dynamics of soil moisture and the EC-derived salinity index. (A–B) Vertical profiles of moisture and salinity for April (Spring, pre-irrigation), July (peak evapotranspiration), and September (Late-season). (C) Temporal evolution of the EC-derived salinity index across the three distinct depth zones. Shaded regions represent the standard error, and the blue vertical band indicates the primary irrigation period.

## Discussion

### Depth-dependent water-salt associations

Soil water-salt relationships vary systematically with depth, driven by balances between evaporative concentration and leaching. Surface layers (0–20 cm) show weak moisture-salinity correlation due to evaporation-dominated dynamics, where capillary rise concentrates salts at the surface [33–36].

Middle layers (20–60 cm) show weak correlations, reflecting dynamic equilibrium between upward and downward fluxes. In subsurface layers (60–100 cm), the weak positive association (R^2^ = 0.06) indicates that moisture is one of multiple interacting factors—including soil hydraulic properties, irrigation history, and groundwater fluctuations—controlling salinity dynamics in these heterogeneous systems.

If the observed relationship were merely a physical artifact of moisture interference on EC sensors, we would expect similar correlation patterns across all depth intervals. However, the data show a depth-dependent divergence: surface layers exhibit essentially no correlation (*r* = 0.07, *p* > 0.05), while deep layers show a statistically significant association (*r* = 0.25, *p* < 0.01). This depth-stratified signal pattern suggests that the deep-layer moisture–salinity association may reflect possible solute transport processes; however, this interpretation remains tentative given the low explanatory power (R^2^ = 0.06) and the observational nature of the dataset, as well as the absence of observations during the peak irrigation period in May. The absence of correlation in surface layers—where moisture effects on EC readings would be most pronounced due to higher variability—further supports the interpretation that deeper correlations represent real redistribution of the EC signal rather than measurement artifacts.

The observed subsurface accumulation pattern (Results, Fig 3) aligns with reported deep-layer salt storage in arid irrigation systems [25,37], and is consistent with potential redistribution patterns; however, these interpretations remain speculative given the weak correlation strength and the low coefficient of determination (R^2^ = 0.06), highlighting the need for multi-season validation, indicating a substantial seasonal accumulation in deep layers that warrants attention for long-term soil quality management. This may indicate potential management considerations under sustained conditions. A potential concern is that these stored salts may return to the rooting zone under dry conditions, as suggested in previous studies [37], although this process warrants further investigation beyond the current single-season dataset. Overall, these depth-dependent associations should be interpreted as indicative patterns rather than evidence of strong mechanistic coupling, particularly given the limited explanatory power of the statistical relationships.

This depth-stratified association pattern is schematically illustrated in Fig 6, which summarises the vertical zonation signals and potential salt transport pathways inferred from the observational data.

**Fig 6:**
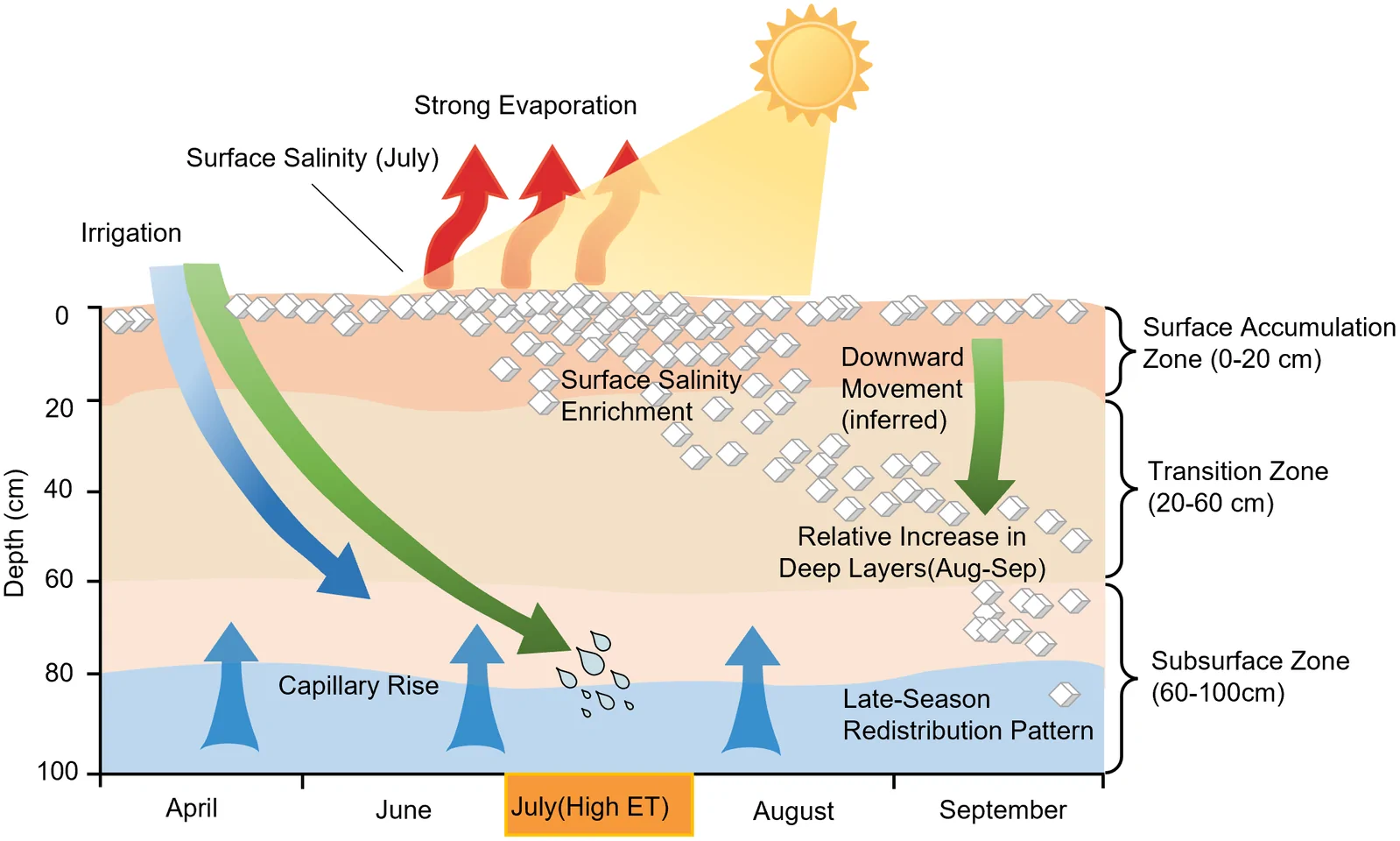
Conceptual model of depth-dependent water–salt redistribution in the Hetao Irrigation District.

### Implications for irrigation management

Although derived from relative EC signals, the observed trend of increasing deep-layer salinity represents a pattern that may warrant management attention. For land managers, ignoring this statistically significant but weak signal could indicate a pattern that merits further monitoring. Our findings are not intended to establish precise predictive models, but rather to demonstrate the necessity of extending monitoring depth to 100 cm as a risk management practice. Even modest subsurface salt loading, if similar patterns persist over multiple seasons, may pose potential risks to soil quality and irrigation effectiveness, although this requires multi-season validation.

The seasonal EC redistribution patterns documented in the Results (Fig 5; Table 3) show that deep-layer ionic loading is not temporally uniform: the most pronounced increase occurred between July and August, coinciding with peak evapotranspiration and reduced irrigation frequency. This timing suggests that drainage operations and late-season irrigation management may represent the most strategically important windows for limiting further subsurface salt accumulation.

This pattern is consistent with the redistribution processes described above, but does not constitute proof of long-term reservoir formation. These patterns reflect relative changes in EC-derived salinity and do not allow assessment of ion-specific risks such as sodicity. Accordingly, given the single-season design and EC-based estimation, the findings should be interpreted as relative dynamics supporting monitoring-based management rather than absolute salt mass assessment.

The application of EC-based estimation enabled rapid, non-destructive salinity assessment across all depths. Based on the observed seasonal patterns (Results, Fig 5), management strategies should prioritize early-season high-volume leaching to prevent mid-season accumulation, while late-season applications should minimize deep percolation to reduce subsurface salt loading.

Because current practices may result in redistribution rather than removal of salts, effective storage capacity may potentially become constrained if similar processes persist over multiple seasons. These dynamics address critical knowledge gaps in understanding complex salt transport dynamics across the vadose zone [24]. Similar subsurface accumulation patterns have been reported in the arid regions of Northwest China and the United States, indicating that deep-layer salt storage is a common yet underreported phenomenon in gravity irrigation systems [25,37]. Our findings suggest depth-differentiated approaches: surface desalination requires high-volume early-season leaching, while late-season applications should minimize deep percolation. The observed subsurface salt loading indicates that drainage infrastructure may be relevant for maintaining soil conditions, suggesting that management attention could be focused on sites where subsurface EC exceeds 800 µS/cm or shows progressive accumulation trends. It should be noted, however, that EC-based thresholds cannot indicate sodicity-related hazards (e.g., sodium adsorption ratio or exchangeable sodium percentage); elevated EC alone does not confirm alkalisation risk without ion-specific analysis. These thresholds are therefore proposed as indicators for prioritising further monitoring, not as criteria for salinity classification or remediation decisions.

Based on these depth-dependent dynamics, Fig 7 presents a practical management framework for optimizing irrigation strategies.

**Fig 7:**
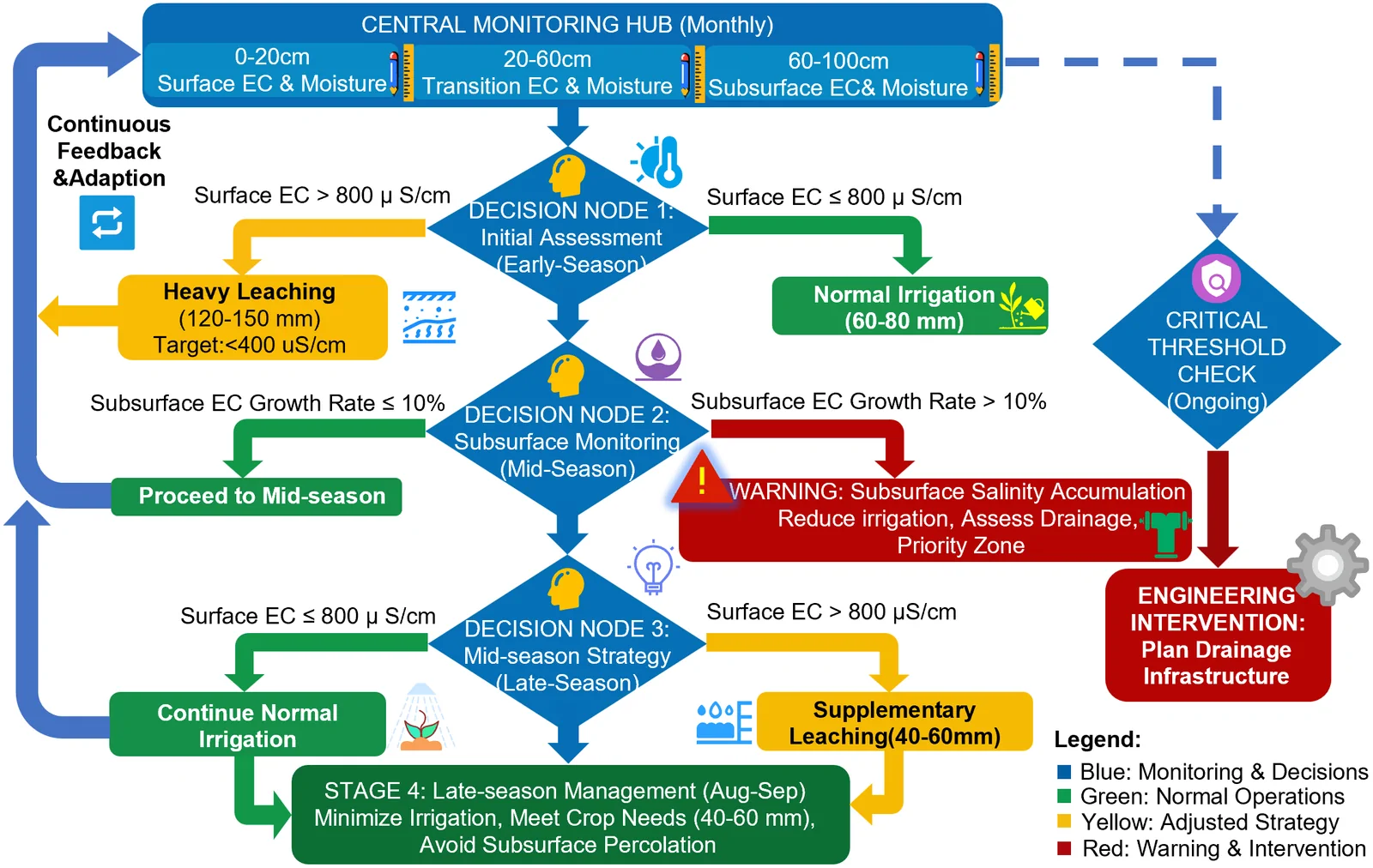
Conceptual framework for adaptive irrigation management based on depth-stratified soil water-salt monitoring.

It should be noted that the threshold values and management responses illustrated in Fig 7 are intended as conceptual guidance based on observed patterns rather than prescriptive criteria, and should be adapted to specific local soil, crop, and hydrological conditions.

### Limitations and future research

Our decision to estimate salt content from EC measurements rather than perform gravimetric analysis on all 450 samples reflects a balance between sampling density and laboratory throughput. The use of linear conversion equations is standard in salinity assessment [28]. Accordingly, the results should be interpreted as reflecting relative salinity variations rather than absolute quantities. Although the use of a fixed regional conversion factor introduces uncertainty compared to direct measurements, it enables the spatiotemporal coverage required to capture EC-derived salinity redistribution patterns.

However, several constraints must be acknowledged. Methodologically, because the salinity index is mathematically derived from EC, our statistical relationships reflect the same underlying ionic signal rather than independent observations. Accordingly, the analysis focuses on qualitative distribution patterns rather than predictive modeling. Furthermore, the sampling design deliberately targeted salinity gradients for process-oriented interpretation; therefore, these results should not be interpreted as regional averages or used to estimate large-scale salt budgets without additional stratified sampling.

Temporally, the reliance on a single growing season restricts the findings to short-term observational evidence. Monthly sampling may miss rapid responses following irrigation events, and the absence of May data—due to continuous irrigation preventing site access—represents a critical data gap during the presumed peak leaching period. Consequently, interpretations of irrigation-driven processes remain inferential. Ultimately, the combination of missing peak-period observations and the lack of multi-season data limits our ability to assess long-term sustainability, highlighting the need for multi-year monitoring to independently validate these depth-dependent redistribution trends.

Future research should prioritize: (1) high-frequency monitoring during peak irrigation events to capture transient leaching dynamics; (2) long-term monitoring to reveal time-to-saturation for salt reservoir capacity; (3) process-based modeling (e.g., HYDRUS, SWAP) to optimize irrigation schedules; (4) coupling field monitoring with emerging remote sensing and machine learning technologies [37–40] to upscale these point-based findings to the district level for precision salinity control, as successfully demonstrated in other large river deltas [41,42]; (5) ion-specific analysis to assess sodicity hazards; and (6) drainage pilot studies to evaluate effectiveness and optimize design parameters.

## Conclusions

This study provides observational evidence of depth-dependent soil water-salt relationships in one of China’s largest irrigation districts. The depth-stratified EC patterns and seasonal dynamics documented in the Results (Figs 1 and 5; Tables 2 and 3) provide observational evidence consistent with subsurface salt redistribution. However, causal inference is constrained by the short-term study design and the absence of May observations. The novelty of this study lies in integrating multi-site full-profile (0–100 cm) observations with a depth-stratified analytical framework under large-scale irrigation conditions. This approach helps reveal subtle depth-dependent signals across the soil profile, although these findings must be interpreted with caution regarding long-term salinity dynamics.

The results indicate that salinity control strategies may benefit from moving from surface-focused leaching to integrated depth-differentiated strategies that consider enhanced drainage infrastructure. Based on the observed seasonal patterns, irrigation timing and volumes should be optimized to balance surface desalination with minimizing subsurface accumulation. Late-season subsurface EC patterns may reflect vertical salt redistribution tendencies. Management attention might therefore be focused on evaluating subsurface drainage needs in areas where EC exceeds 800 µS/cm.

These findings highlight limitations of conventional leaching-based management approaches that primarily focus on surface salt removal while overlooking subsurface redistribution. The observed salinity patterns provide field-based indications of how salts may be redistributed within the soil profile under large-scale irrigation, contributing to our understanding of salinization dynamics in the Hetao Irrigation District and potentially in other arid irrigation systems with similar conditions. From a management perspective, the results underscore the importance of considering strategies that facilitate effective salt removal from the soil profile, rather than approaches that predominantly relocate salts to deeper layers over time.

## Supporting information

S1 Dataset. Raw data. The complete dataset of soil moisture, EC, and salt content used in this study.
(CSV)
