# Supplementary material for: Depth-dependent soil water-salt relationships in the Hetao Irrigation District, arid Northwest China: Implications for sustainable management
Source: PLoS One. 2026 Oct 20;21(10):e0360976. doi: 10.1371/journal.pone.0360976 (PMC13682659; doi:10.1371/journal.pone.0360976)
Supplement: Supplementary file 1 [file pone.0360976.s003.pdf]

PLOS One

Depth-dependent soil water-salt relationships in the Hetao Irrigation District, arid Northwest China: Implications for sustainable management

--Manuscript Draft--

**Manuscript Number:**	PONE-D-26-08043
**Article Type:**	Research Article
**Full Title:**	Depth-dependent soil water-salt relationships in the Hetao Irrigation District, arid Northwest China: Implications for sustainable management
**Short Title:**	Depth-dependent soil water-salt relationships
**Corresponding Author:**	Xuan Chen<br>Hetao College<br>Bayannur, CHINA
**Keywords:**	soil salinity; electrical conductivity; irrigation management; vertical distribution; Hetao irrigation district
**Abstract:**	Soil salinization threatens agricultural productivity in arid gravity irrigation systems worldwide, yet the depth-stratified relationships between soil water and salt content remain poorly characterized. We monitored soil moisture, electrical conductivity (EC), and salt content at six depths (0–100 cm) across 15 sites in the Hetao Irrigation District, China, during the 2025 growing season. Our results indicate a depth-dependent decoupling of water-salt dynamics. EC-derived soil salt content varied from 0.08 to 42.91 g/kg, exhibiting extreme spatial heterogeneity (coefficient of variation (CV) = 185.2%) across the salinity gradient. However, moisture-salinity relationships showed marked depth dependency. Surface soils (0–20 cm) exhibited non-statistically significant correlations ($r = 0.07$), whereas deep layers (60–100 cm) showed a statistically significant but weak positive correlation ($r = 0.25$, $p < 0.01$, $R^2 = 0.06$; small effect size), consistent with patterns of downward salt migration, suggesting deep layers may act as temporary sinks. The EC-derived salinity index showed a 42% increase in deep layers (from 1.59 to 2.26 g/kg) over the season, compared to a 136% fluctuation in surface layers. These results highlight potential challenges of surface-focused monitoring approaches and indicate patterns consistent with the hypothesis of subsurface salt loading, representing a potential constraint on the long-term effectiveness of leaching-based salinity management. The dataset and findings provide observational insights for informing depth-differentiated irrigation strategies and supporting salinity management in arid agricultural systems.
**Order of Authors:**	Yuanqing Cao
	Huihui Wang
	Xuan Chen
**Additional Information:**	
**Question**	**Response**
**Financial Disclosure**<br><br>Enter a financial disclosure statement that describes the sources of funding for the work included in this submission. Review the submission guidelines for detailed requirements. View published research articles from *PLOS ONE* for specific examples.<br><br>This statement is required for submission	Yes

Powered by Editorial Manager® and ProduXion Manager® from Aries Systems Corporation

and **will appear in the published article** if the submission is accepted. Please make sure it is accurate.<br><br>**Funded studies**<br>Enter a statement with the following details:<br>• Initials of the authors who received each award<br>• Grant numbers awarded to each author<br>• The full name of each funder<br>• URL of each funder website<br>• Did the sponsors or funders play any role in the study design, data collection and analysis, decision to publish, or preparation of the manuscript?<br><br>Did you receive funding for this work?	
Please add funding details.<br>as follow-up to "**Financial Disclosure**<br><br>Enter a financial disclosure statement that describes the sources of funding for the work included in this submission. Review the submission guidelines for detailed requirements. View published research articles from *PLOS ONE* for specific examples.<br><br>This statement is required for submission and **will appear in the published article** if the submission is accepted. Please make sure it is accurate.<br><br>**Funded studies**<br>Enter a statement with the following details:<br>• Initials of the authors who received each award<br>• Grant numbers awarded to each author<br>• The full name of each funder<br>• URL of each funder website<br>• Did the sponsors or funders play any role in the study design, data collection and analysis, decision to publish, or preparation of the manuscript?<br><br>Did you receive funding for this work?"	This work was supported by the Science and Technology Project of Bayannur City, Inner Mongolia Autonomous Region (titled "Key Parameters Sensing and Intelligent Platform for Agricultural Hydrological Processes in Irrigation Districts", Grant No. NMKJXM202407) awarded to Y.C.. The funders had no role in study design, data collection and analysis, decision to publish, or preparation of the manuscript.

Please select the country of your main research funder (please select carefully as in some cases this is used in fee calculation).<br>as follow-up to "**Financial Disclosure**<br><br>Enter a financial disclosure statement that describes the sources of funding for the work included in this submission. Review the submission guidelines for detailed requirements. View published research articles from *PLOS ONE* for specific examples.<br><br>This statement is required for submission and **will appear in the published article** if the submission is accepted. Please make sure it is accurate.<br><br>**Funded studies**<br>Enter a statement with the following details:<br>• Initials of the authors who received each award<br>• Grant numbers awarded to each author<br>• The full name of each funder<br>• URL of each funder website<br>• Did the sponsors or funders play any role in the study design, data collection and analysis, decision to publish, or preparation of the manuscript?<br><br>Did you receive funding for this work?"	CHINA - CN
**Competing Interests**<br><br>Use the instructions below to enter a competing interest statement for this submission. On behalf of all authors, disclose any competing interests that could be perceived to bias this work—acknowledging all financial support and any other relevant financial or non-financial competing interests.<br><br>This statement is required for submission and **will appear in the published article** if the submission is accepted. Please make sure it is accurate and that any funding	The authors have declared that no competing interests exist.

sources listed in your Funding Information later in the submission form are also declared in your Financial Disclosure statement.<br><br>View published research articles from *PLOS ONE* for specific examples.<br><br>**NO authors have competing interests**<br><br>Enter: *The authors have declared that no competing interests exist.*<br><br>**Authors with competing interests**<br><br>Enter competing interest details beginning with this statement:<br><br>*I have read the journal's policy and the authors of this manuscript have the following competing interests: [insert competing interests here]*<br><br>* typeset	
**Ethics Statement**<br><br>Enter an ethics statement for this submission. This statement is required if the study involved:<br><br>• Human participants<br>• Human specimens or tissue<br>• Vertebrate animals or cephalopods<br>• Vertebrate embryos or tissues<br>• Field research<br><br>Write "N/A" if the submission does not require an ethics statement.<br><br>General guidance is provided below. Consult the submission guidelines for detailed instructions. **Make sure that all information entered here is included in the Methods section of the manuscript.**	N/A

**Format for specific study types**<br><br>**Human Subject Research (involving human participants and/or tissue)**<br>• Give the name of the institutional review board or ethics committee that approved the study<br>• Include the approval number and/or a statement indicating approval of this research<br>• Indicate the form of consent obtained (written/oral) or the reason that consent was not obtained (e.g. the data were analyzed anonymously)<br><br>**Animal Research (involving vertebrate animals, embryos or tissues)**<br>• Provide the name of the Institutional Animal Care and Use Committee (IACUC) or other relevant ethics board that reviewed the study protocol, and indicate whether they approved this research or granted a formal waiver of ethical approval<br>• Include an approval number if one was obtained<br>• If the study involved *non-human primates*, add *additional details* about animal welfare and steps taken to ameliorate suffering<br>• If anesthesia, euthanasia, or any kind of animal sacrifice is part of the study, include briefly which substances and/or methods were applied<br><br>**Field Research**<br><br>Include the following details if this study involves the collection of plant, animal, or other materials from a natural setting:<br>• Field permit number<br>• Name of the institution or relevant body that granted permission	
**Data Availability**<br><br>Authors are required to make all data underlying the findings described fully available, without restriction, and from the time of publication. PLOS allows rare exceptions to address legal and ethical concerns. See the PLOS Data Policy and FAQ for detailed information.	Yes - all data are fully available without restriction

A Data Availability Statement describing where the data can be found is required at submission. Your answers to this question constitute the Data Availability Statement and **will be published in the article**, if accepted.<br><br>**Important:** Stating 'data available on request from the author' is not sufficient. If your data are only available upon request, select 'No' for the first question and explain your exceptional situation in the text box.<br><br>Do the authors confirm that all data underlying the findings described in their manuscript are fully available without restriction?	
**Describe where the data may be found in full sentences. If you are copying our sample text, replace any instances of XXX with the appropriate details.**<br><br>• If the data are **held or will be held in a public repository**, include URLs, accession numbers or DOIs. If this information will only be available after acceptance, indicate this by ticking the box below. For example: *All XXX files are available from the XXX database (accession number(s) XXX, XXX.).*<br>• If the data are all contained **within the manuscript and/or Supporting Information files**, enter the following: *All relevant data are within the manuscript and its Supporting Information files.*<br>• If neither of these applies but you are able to provide **details of access elsewhere**, with or without limitations, please do so. For example:<br><br>*Data cannot be shared publicly because of [XXX]. Data are available from the XXX Institutional Data Access / Ethics Committee (contact via XXX) for researchers who meet the criteria for access to confidential data.*<br><br>*The data underlying the results presented in the study are available from (include the name of the third party*	All relevant data are within the manuscript and its Supporting Information files.

and contact information or URL).<br>• This text is appropriate if the data are owned by a third party and authors do not have permission to share the data.<br><br>* typeset	
Additional data availability information:	

Click here to access/download;Manuscript;Manuscript.docx

Depth-dependent soil water-salt relationships in the Hetao Irrigation District, arid Northwest China: Implications for sustainable management

Short Title: Depth-dependent soil water-salt relationships

Yuanqing Cao[1], Huihui Wang[1], Xuan Chen[2*]

[1] Department of Mathematics and Computer Science, Hetao College, Bayannur, Inner Mongolia, China

[2] Department of Hydraulic and Civil Engineering, Hetao College, Bayannur, Inner Mongolia, China

[*] Corresponding author

E-mail: 412766722@qq.com (XC)

Abstract

Soil salinization threatens agricultural productivity in arid gravity irrigation systems worldwide, yet the depth-stratified relationships between soil water and salt content remain poorly characterized. We monitored soil moisture, electrical conductivity (EC), and salt content at six depths (0–100 cm) across 15 sites in the Hetao Irrigation District, China, during the 2025 growing season. Our results indicate a depth-dependent decoupling of water-salt dynamics. EC-derived soil salt content varied from 0.08 to 42.91 g/kg, exhibiting extreme spatial heterogeneity (coefficient of variation (CV) = 185.2%) across the salinity gradient. However, moisture-salinity relationships showed marked depth dependency. Surface soils (0–20 cm) exhibited non-statistically significant correlations (r = 0.07), whereas deep layers (60–100 cm) showed a statistically significant but weak positive correlation (r = 0.25, p < 0.01, R^2 = 0.06; small effect size), consistent with patterns of downward salt migration, suggesting deep layers may act as temporary sinks. The EC-derived salinity index showed a 42% increase in deep layers (from 1.59 to 2.26 g/kg) over the season, compared to a 136% fluctuation in surface layers. These results highlight potential challenges of surface-focused monitoring approaches and indicate patterns consistent with the hypothesis of subsurface salt loading, representing a potential constraint on the long-term effectiveness of leaching-based salinity management. The dataset and findings provide observational insights for

informing depth-differentiated irrigation strategies and supporting salinity management in arid agricultural systems.

Introduction

Soil salinization affects ~20% of irrigated lands worldwide, expanding at 1-2 million hectares annually [1-6]. In China, 36 million hectares are salt-affected [7], with the Hetao Irrigation District—one of China's three largest irrigation districts—exemplifying this crisis: over 60% of its area suffers irrigation-induced salinity [8,9], threatening long-term productivity.

Understanding soil water-salt relationships is fundamental to managing salinization, as water movement governs salt transport through upward capillary flow (evaporation-driven) and downward leaching (irrigation/precipitation-induced) [10,11]. These fluxes create complex spatial and temporal salt distribution patterns varying with depth, texture, groundwater, and management [12]. However, most studies focus on surface soil (0–20 cm) or assume uniform profiles, missing critical deeper-layer dynamics where salts accumulate during leaching and redistribute later [13]. Deep-layer salt accumulation challenges long-term sustainability: subsurface layers may approach storage limits under sustained irrigation, reducing leaching effectiveness and increasing upward redistribution to roots [4,5]. This may increase irrigation demand and exacerbate salinity management challenges [14].

Electrical conductivity (EC) offers rapid salinity assessment [15,16], though moisture effects and depth-dependent ionic variations require careful calibration [17-20].

Seasonal dynamics add complexity to salinity management. In gravity-fed systems like Hetao, managed flooding (May-July) leaches salts [21], but effectiveness varies with climate and depth. Traditional approaches assume sufficient leaching fraction will maintain acceptable salinity levels by flushing salts downward [22]. However, irrigation may redistribute salts deeper rather than removing them [23], and vadose zone salt storage constrains long-term leaching effectiveness [24,25]. This raises fundamental questions about long-term sustainability and the ultimate fate of leached salts. This concerns regions like Hetao, where shallow groundwater tables (1.5–3.5 m) may intercept downward salts and create reservoirs that return to the profile through capillary rise.

This study systematically analyzes depth-stratified water-salt relationships across a representative 150 km² area of the Hetao Irrigation District. We integrate field monitoring with statistical modeling to: (1) characterize spatiotemporal heterogeneity of electrical conductivity (EC)-based salinity from surface (0–20 cm) to deep (60–100 cm) layers; (2) examine depth-dependent moisture-salinity coupling; (3) identify temporal salt redistribution patterns. Unlike surface-focused studies, this work provides comprehensive vertical salt

distribution data, offering insights for sustainable salinity management in arid irrigation systems.

Materials and methods

Study area

The Hetao Irrigation District (40°13′–41°16′N, 106°20′–109°19′E) occupies 574,000 ha in Inner Mongolia, northwestern China, between the Yellow River (south) and Yinshan Mountains (north). The region has a temperate continental monsoon climate: cold winters and hot, dry summers. Mean annual temperature is 6.8°C (January: −11.6°C; July: 23.2°C). Annual precipitation averages 139 mm (range: 130–215 mm), concentrated July–September, while potential evapotranspiration reaches 2,200 mm, creating severe moisture deficits requiring intensive irrigation.

The irrigation system diverts 4–5 billion m^3 annually from the Yellow River through over 10,000 km of canals. Irrigation (April–September) typically applies 450–600 mm across 8–12 events depending on crop requirements and soil conditions. The drainage system comprises 15 primary channels (807 km total) plus secondary/tertiary networks, but capacity remains insufficient in many areas, contributing to shallow groundwater and salinization [26].

Soils are predominantly alluvial deposits from Yellow River sediments, classified as Aridisols and Entisols (USDA taxonomy). Textures range from sandy loam to silt loam, with low organic matter (8–15 g/kg) typical of arid soils.

Bulk density averages 1.35–1.45 g/cm³; hydraulic conductivity varies 0.5–5 cm/h by texture. Groundwater depth varies 1.5–3.5 m, fluctuating 0.5–1.0 m seasonally with irrigation and drainage.

The dominant cropping pattern is spring wheat (April–July) followed by sunflower (July–October), though maize and other crops occur locally. This double-cropping system maximizes productivity but intensifies water demand and complicates salinity management by requiring irrigation when evaporative demand peaks [27].

Sampling design

We established 15 monitoring sites (spring 2025) capturing spatial salinity heterogeneity across representative farmland. Sites were selected via stratified sampling based on preliminary surveys with a portable salinity sensor (TZS-ECW, Zhejiang Top Cloud-agri Technology, China). This process deliberately included locations from non-saline to highly saline, preventing low-salinity bias. This design targeted gradient analysis rather than regional averages.

All sites had wheat-sunflower rotation with similar management. Sites spaced 0.5–8 km across ~150 km². Based on these preliminary survey values, the 15 monitoring sites were initially categorized into four salinity classes to maximize gradient coverage: non-saline (EC < 400 μS/cm, 7 sites), slightly saline (EC 400–1000 μS/cm, 3 sites), moderately saline (EC 1000–2000 μS/cm, 2 sites), and highly saline (EC > 2000 μS/cm, 3 sites).

Sampling occurred monthly April 15–September 15, 2025 (excluding May). For consistency, samples were collected days 15–20 of each month, ≥5 days after irrigation or rainfall. This timing minimizes transient moisture effects while reflecting typical growth-period conditions. May was excluded due to continuous irrigation preventing site access. The dataset comprises April, June, July, August, and September measurements.

At each site, we sampled six depths: 0–10, 10–20, 20–40, 40–60, 60–80, and 80–100 cm. These intervals capture near-surface dynamics (0–20 cm), active rooting zone (20–60 cm), and deeper salt sinks (60–100 cm). Three replicate cores within a 2-m radius were extracted using a stainless-steel auger (Type TY-1, Nanjing Soil Instrument Factory, China; 3 cm diameter, 100 cm length) and composited by depth. Each depth yielded 500–800 g soil. Samples were sealed in labeled bags and transported within 6 hours. This yielded 450 samples (15 sites × 6 depths × 5 months). Permanent markers ensured consistent sampling locations.

Laboratory analyses

In the laboratory, samples were divided into subsamples. One subsample determined moisture: field-moist samples were weighed, oven-dried (105°C, 24 h to constant weight), and re-weighed for gravimetric water content. Remaining soil was air-dried (7–10 days), ground through a 2-mm sieve, and stored for analysis.

Electrical conductivity ($EC_{1:5}$) was measured in 1:5 soil: water extracts following standard protocols [28]. Air-dried soil (20 g) was mixed with deionized water (100 mL), shaken (5 min), settled (30 min), and filtered (quantitative filter paper, slow speed, Great Wall, China). EC was measured at 25°C (DDS-307A meter, Rex Electric Chemical, Shanghai; ±1% accuracy).

Soil salt content (SSC) was estimated from $EC_{1:5}$ measurements using a published conversion equation calibrated for Hetao Irrigation District soils [29]:

$$SSC\ (g/kg) = 0.0037657 \times EC1{:}5\ (\mu S/cm) - 0.2405 \quad (1)$$

The objective is to examine how soil moisture dynamics are associated with the spatial heterogeneity and profile redistribution of EC-derived salinity indices, rather than to estimate absolute salt mass or establish strong causal relationships. This approach prioritizes pattern recognition in field monitoring data over mechanistic modeling, recognizing that weak correlations (e.g., R^2 = 0.06) reflect the complex, multifactorial nature of salinity dynamics in heterogeneous arid systems.

The conversion equation derives from linear regression between $EC_{1:5}$ and gravimetric salts (regional calibration), applied as a practical conversion tool rather than predictive model.

Where SSC = soil salt content (g/kg) and $EC_{1:5}$ = electrical conductivity (μS/cm). This relationship was developed from Hetao field measurements and validated in regional studies [29,30]. This EC conversion approach is widely

adopted for rapid, non-destructive salt estimation [16,28]. While this regional calibration provides consistent relative estimates, site-specific calibration might improve absolute accuracy. Therefore, values represent EC-derived relative patterns rather than precise gravimetric measurements.

Quality control included duplicate samples (~10%). Relative percent differences averaged 4.2% for moisture, 5.8% for EC, and 6.3% for salt content—all within acceptable ranges. Instruments were daily-calibrated using standard KCl.

Data analysis

Descriptive statistics (mean, standard deviation (SD), CV, range) were calculated for moisture, EC, and salt content ($n = 450$), stratified by depth or month. Data distributions were examined using histograms and quantile–quantile plots. Although EC and salt content were right-skewed, Pearson correlation was appropriate given sample size and robustness to moderate non-normality.

Pearson correlations quantified relationships among moisture, EC, and salt content for the full dataset and three depth classes: surface (0–20 cm), transition (20–60 cm), subsurface (60–100 cm). Spearman rank correlations confirmed robustness to non-normality. Significance was evaluated at $\alpha = 0.05$; effect sizes followed Cohen's guidelines [31] (small: $r \leq 0.10$; medium: $r \leq 0.30$; large: $r > 0.50$).

Multiple linear regression examined relationships between salt content and explanatory variables (EC, moisture, depth). Model performance used R^2 and root mean square error (RMSE). Assumptions were assessed via residual diagnostics; stability via leave-one-out cross-validation.

Temporal variations used time-series visualizations and analysis of variance (ANOVA), with site as a blocking factor for repeated measurements. Pairwise monthly differences used Tukey's Honestly Significant Difference (HSD) test.

To overcome the limitation of weak linear correlation ($r = 0.25$, $R^2 = 0.06$) and to avoid circular regression, a group-comparison strategy was adopted as recommended for pattern recognition in heterogeneous field data. Soil samples were stratified by (1) depth intervals (0–20 cm: surface; 20–60 cm: transition; 60–100 cm: deep), and (2) moisture classes defined by empirical terciles of the dataset within each depth category (Low: ≤33rd percentile; Medium: 33rd–67th percentile; High: >67th percentile).

Differences in EC among moisture classes within each depth were evaluated using one-way ANOVA with Bonferroni-corrected pairwise t-tests ($\alpha = 0.05/3 = 0.0167$). This approach shifts the analytical focus from regression prediction to response pattern analysis—examining how EC signals vary across moisture gradients rather than attempting to predict absolute salt mass from moisture.

Box plots were generated to visualize EC distributions across moisture classes for each depth category. This group-comparison approach allows identification of consistent EC-moisture associations even when overall linear correlations are weak, as is expected in complex, multifactorial arid soil systems where salinity dynamics are influenced by numerous interacting processes (irrigation timing, evaporation, capillary rise, soil texture heterogeneity).

All analyses used Python 3.13.11 (NumPy, Pandas, SciPy, statsmodels) [32]. Because salt content derives from EC, regression analyses evaluate how moisture and depth modify the EC-derived signal, not predict salt from EC (circular). High R^2 values reflect the deterministic conversion, serving as consistency checks rather than predictive performance.

Results

Spatial variability and general characteristics

Unless otherwise specified, all SSC values reported in this section are estimates derived from $EC_{1:5}$ measurements using Eq. 1.

Soil properties exhibited substantial spatial heterogeneity (Table 1). Gravimetric moisture averaged 0.157 g/g but varied widely (range 0.005–0.442, CV = 49%), reflecting soil texture, irrigation, weather, and groundwater depth. The extremely low minimum value (0.005 g/g) represents near-air-dry conditions in surface soils during hot, dry periods between irrigations, while the maximum (0.442 g/g) occurred in fine-textured soils shortly after irrigation.

Electrical conductivity showed extreme variability (CV = 167.4%), ranging nearly three orders of magnitude from 85 to 11,460 μS/cm. Similarly, salt content spanned from 0.08 to 42.91 g/kg (mean = 2.26 g/kg, CV = 185.2%), indicating the presence of both non-saline and severely salt-affected locations within the study area.

Table 1. Summary statistics for soil properties across all sampling sites, depths, and months (n = 450).

Property	Mean	SD	Min	Max	CV (%)
**Soil Moisture (g/g)**	0.16	0.08	0.00	0.44	49.0
**EC (μS/cm)**	663.81	1111.39	85.00	11460.00	167.4
**Salt Content (g/kg)**	2.26	4.19	0.08	42.91	185.2

The spatial distribution of soil salinity across the 15 sites revealed distinct potential zones of salt redistribution that appear to be controlled by topography, drainage infrastructure, and groundwater conditions. Three sites (YG6, YG9, YG13) consistently exhibited high EC values (>1000 μS/cm on average), suggesting chronic salinization problems potentially linked to inadequate drainage, proximity to saline groundwater, or accumulation in topographic depressions. These sites showed the highest spatial variability, with coefficients of variation exceeding 200%. Conversely, seven sites (YG1, YG2, YG3, YG8, YG10, YG14, YG15) maintained relatively low salinity (EC < 400 μS/cm) throughout the season, demonstrating effective natural or managed drainage. The remaining sites showed intermediate or variable conditions, with salinity

levels fluctuating seasonally in response to irrigation and evapotranspiration cycles.

Depth-stratified moisture-salinity relationships

The dataset exhibited a highly skewed salinity distribution, with values ranging from 0.08 to 42.91 g/kg, reflecting the extreme spatial heterogeneity typical of arid irrigation districts. Depth-stratified analysis reveals distinct moisture-salinity coupling patterns across the soil profile. Surface layers (0–20 cm) exhibited weak and non-statistically significant correlations between soil moisture and estimated salt content ($r = 0.07$, $P > 0.05$), indicating that surface salinity dynamics are driven principally by rapid evaporation and irrigation pulses rather than consistent moisture-driven leaching. In contrast, the moisture-salinity relationship shifted significantly with depth. The transition zone (20–60 cm) showed intermediate behavior ($r = 0.12$), reflecting a dynamic equilibrium. Critically, the deep subsurface layers (60–100 cm) showed a statistically significant but weak positive correlation ($r = 0.25$, $P < 0.01$; small effect size). This weak association suggests a tendency toward moisture-salinity coupling in these deep layers rather than strong mechanistic control, where moisture conditions significantly alter the probability distribution of deep-layer salinity resulting from long-term downward leaching. These depth-stratified patterns are illustrated in Fig 1.

Fig 1. Statistical characteristics and water-salt coupling relationships across the soil profile. (A) Frequency distribution of estimated salt content (n =450), exhibiting high skewness (Mean = 2.26 g/kg, Max = 42.91 g/kg). (B–D) Moisture-Salt Relationships by Depth: (B) Surface (0–20 cm) shows no significant correlation (r = 0.07, p > 0.05); (C) Transition (20–60 cm) shows intermediate coupling (r = 0.12); (D) Subsurface (60–100 cm) reveals a significant positive correlation (r = 0.25, p < 0.01), consistent with deep-layer retention. Solid lines indicate linear regression fits with 95% confidence intervals (shaded).

The moisture-class stratification results are presented in Fig 2 and provide additional insight into EC patterns across the soil profile. One-way ANOVA revealed statistically significant differences in EC among moisture classes for all three depth categories (Surface: F = 3.42, p = 0.035; Transition: F = 3.94, p = 0.022; Deep: F = 4.38, p = 0.014; Fig 2). In the deep layers (60–100 cm), pairwise comparisons showed that both Medium and High moisture classes had significantly higher EC values than the Low moisture class (p < 0.01 after Bonferroni correction), with mean differences of 411 μS/cm and 291 μS/cm, respectively. This pattern is consistent with, but does not definitively prove, the hypothesis that irrigation leaching redistributes EC signals toward deeper soil layers. Importantly, these group-level differences remain detectable even though the overall linear correlation is weak (r = 0.25, R^2 = 0.06), highlighting

that moisture-driven patterns remain statistically detectable despite high field heterogeneity that obscures simple linear relationships, demonstrating that meaningful EC-moisture associations can exist in complex field systems despite low explained variance in regression models.

Fig 2. EC distribution across moisture classes by depth category. Box plots display median (line), IQR (box), whiskers (1.5×IQR), and outliers (gray points); red diamonds indicate means. ANOVA confirms significant differences at all depths ($p < 0.05$). Crucially, in deep layers (60–100 cm), Medium and High moisture classes exhibit significantly elevated EC compared to Low moisture ($p < 0.01$, Bonferroni-corrected), consistent with leaching-driven downward redistribution.

Average soil properties varied systematically with depth (Table 2). Moisture content increased progressively from 0.129 g/g in surface soils to 0.190 g/g at 80–100 cm depth, consistent with typical evaporative gradients in arid-zone profiles.

Table 2. Mean soil properties by depth interval averaged across all sites and sampling months.

Depth (cm)	Moisture (g/g)	EC (µS/cm)	Salt Content (g/kg)
**0-10**	0.129 ± 0.071	980.3 ± 1810.5	3.45 ± 6.82
**10-20**	0.135 ± 0.068	731.2 ± 1396.5	2.51 ± 5.26
**20-40**	0.145 ± 0.065	598.7 ± 847.2	2.01 ± 3.19
**40-60**	0.170 ± 0.079	538.8 ± 574.6	1.79 ± 2.16
**60-80**	0.175 ± 0.076	546.7 ± 705.4	1.82 ± 2.66

**80-100**	0.190 ± 0.084	587.1 ± 758.2	1.97 ± 2.86

Electrical conductivity and salt content showed more complex patterns, with maximum values in surface layers (0–10 cm: EC = 980 μS/cm, *salt* = 3.45 g/kg), decreasing to minima around 20–40 cm depth (EC = 599 μS/cm, *salt* = 2.01 g/kg), then increasing again in deeper layers (80–100 cm: EC = 587 μS/cm, *salt* = 1.97 g/kg). This bimodal salinity distribution—with peaks at both surface and depth—indicates dual accumulation mechanisms. Surface enrichment results from evaporative concentration, well-documented in arid agriculture. The deeper accumulation represents a notable management concern: it suggests that current irrigation practices may be associated with patterns consistent with subsurface salt loading, inferred from downward EC signal changes during leaching events, potentially indicating accumulation above a hydraulic boundary that impedes further movement.

The depth of minimum salinity (20–40 cm) may represent a notable zone where irrigation leaching is most effective, located deep enough to escape intense evaporative enrichment but shallow enough to receive regular leaching from irrigation water percolating downward.

Seasonal dynamics across depth zones

Having established the depth-dependent moisture-salinity relationships, we now examine how these patterns evolve temporally over the growing season. The irrigation-induced salt redistribution processes operate at different timescales across

soil depths, creating complex spatiotemporal dynamics that require careful monitoring.

The spatiotemporal distribution of soil salinity across the growing season is visualized in Fig 3, which provides a comprehensive heatmap showing the temporal evolution of salt content at different depths from April to September. This visualization reveals distinct accumulation patterns in three functional zones of the soil profile.

Fig 3. Spatiotemporal distribution of soil salt content. Heatmap visualization (Apr–Sep 2025) where warm colors indicate high salinity. The profile reveals: (1) persistent surface accumulation (0–20 cm) with net seasonal increase of 136% from April (1.30 g/kg) to September (3.06 g/kg), peaking at 5.57 g/kg in July; (2) a dynamic transition zone (20–60 cm) (+55%); and (3) progressive subsurface loading (60–100 cm) with a 42% increase (1.59 to 2.26 g/kg), consistent with downward salt redistribution.

The spatiotemporal distribution pattern illustrates these depth-dependent dynamics across the growing season.

Seasonal patterns revealed substantial temporal variability (Fig 4, Table 3). Moisture content peaked in June (0.192 g/g) during the main irrigation period, declined through July–August (0.143–0.160 g/g) as crop water uptake and evapotranspiration exceeded inputs, and reached annual lows in September (0.136 g/g).

Fig 4. Seasonal dynamics of soil properties. Monthly changes in moisture (blue), salt content (red), and EC (green) averaged across all sites (April–September 2025); shaded regions represent standard error. Moisture peaked in June (0.192 g/g) during intensive irrigation, subsequently declining to September (0.136 g/g). In contrast, salinity and EC exhibited a lagged peak, rising slightly in June (1.45 g/kg) before surging to a maximum of 3.21 g/kg (EC: 917 μS/cm) in July driven by evapotranspiration, then moderating to 2.44 g/kg in September.

Table 3. Monthly averages of soil properties across all sites and depths.

Month	Moisture (g/g)	EC (μS/cm)	Salt Content (g/kg)
**April**	0.156 ± 0.083	433.1 ± 435.0	1.39 ± 1.64
**June**	0.192 ± 0.082	448.8 ± 263.7	1.45 ± 0.99
**July**	0.143 ± 0.054	916.7 ± 1833.5	3.21 ± 6.90
**August**	0.160 ± 0.084	808.8 ± 1145.2	2.81 ± 4.31
**September**	0.136 ± 0.066	711.6 ± 1053.9	2.44 ± 3.97

Salinity showed inverse trends. The lowest values occurred in April (1.39 g/kg) following winter snowmelt. Salt content increased during June (1.45 g/kg) despite heavy irrigation, then peaked in July (3.21 g/kg) when hot, dry conditions drove intense evapotranspiration. A modest decline in August–September (2.81–2.44 g/kg) corresponded with reduced evaporative demand.

Surface soils (0-20 cm) showed dramatic variability: salinity increased 21% from April to June despite irrigation, then surged 329% by July, reflecting evaporative dominance during peak summer heat.

Subsurface layers (60–100 cm) exhibited complex seasonal patterns: salinity decreased slightly from April (1.59 g/kg) to June (1.47 g/kg), remained relatively stable through July (1.47 g/kg), then increased dramatically in August (2.65 g/kg) due to late-season salt redistribution, before declining moderately to September (2.26 g/kg). The overall seasonal increase of 42% from April to September indicates that irrigation leaching is associated with seasonal salt redistribution into deeper layers rather than complete removal from the system. This subsurface accumulation represents a long-term threat: salts stored at 60–100 cm depth remains available for upward transport during dry periods.

To provide detailed quantitative analysis of these seasonal patterns, Fig 5 presents vertical profiles and temporal evolution curves for both moisture and salt content across the three depth zones. This detailed view reveals the specific timing and magnitude of salt redistribution events, showing three distinct zonal behaviors across the soil profile.

Fig 5. Seasonal dynamics of soil moisture and EC-derived soil salt content. (A–B) Vertical Profiles: Moisture and salt profiles for April (pre-irrigation), July (peak ET), and September. Salt distribution diverges from uniform April conditions (~1.3 g/kg) to a sharp surface peak (5.57 g/kg) in July. (C) Temporal Evolution: Three distinct zonal behaviors are evident. The Surface Zone (0–20 cm, red) exhibited extreme fluctuation, surging 329% to a July peak; the Transition Zone (orange) showed intermediate modulation; while

the Subsurface Zone (60–100 cm, blue) demonstrated progressive accumulation (+42%), peaking later in August (2.65 g/kg) consistent with downward salt migration. Shaded regions represent variability; the blue band marks the irrigation period. Note: Data for May were not collected due to field irrigation events.

Discussion

Depth-dependent water-salt coupling

Soil water-salt relationships vary systematically with depth, driven by balances between evaporative concentration and leaching. Surface layers (0-20 cm) show weak moisture-salinity correlation due to evaporation-dominated dynamics, where capillary rise concentrates salts at the surface [33-36].

Middle layers (20-60 cm) show weak correlations, reflecting dynamic equilibrium between upward and downward fluxes. Subsurface layer dynamics (60–100 cm) show a statistically significant but weak positive moisture-salinity correlation ($r = 0.25$, $R^2 = 0.06$; small effect size), suggesting that these layers may act as potential zones of salt redistribution under irrigation-driven leaching, where downward salt transport exceeds removal from the soil profile. The bimodal salinity distribution corroborates this interpretation.

The weak correlation ($R^2 = 0.06$) reflects the multifactorial nature of salinity dynamics in heterogeneous systems, where moisture is one of many interacting factors including soil hydraulic properties, ionic composition, irrigation history,

and groundwater fluctuations. If the observed relationship were merely a physical artifact of moisture interference on EC sensors, we would expect similar correlation patterns across all depth intervals. However, the data show a depth-dependent divergence: surface layers exhibit essentially no correlation ($r = 0.07$, $p > 0.05$), while deep layers show a statistically significant association ($r = 0.25$, $p < 0.01$). This depth-stratified signal pattern suggests that the deep-layer moisture-salinity coupling reflects genuine solute transport processes rather than systematic sensor error. The absence of correlation in surface layers—where moisture effects on EC readings would be most pronounced due to higher variability—further supports the interpretation that deeper correlations represent real salt redistribution rather than measurement artifacts.

The observed subsurface accumulation pattern (Results, Fig 3) aligns with reported deep-layer salt storage in arid irrigation systems [25,37], indicating a substantial seasonal accumulation in deep layers that warrants attention for long-term soil quality management. The observed pattern indicates a potential risk that continued accumulation could pose long-term challenges to soil salinity management if similar irrigation and transport conditions persist over multiple growing seasons. More concerning is the potential for these stored salts to return to the rooting zone during dry periods, negating years of leaching efforts through salt cycling [37].

This depth-dependent coupling mechanism is conceptualized in Fig 6, which synthesizes the vertical zonation patterns and major salt transport pathways observed in this study.

Fig 6. Conceptual model of depth-dependent water and salt dynamics in the Hetao Irrigation District. The diagram illustrates three functional zones with distinct temporal characteristics: (1) Surface Accumulation Zone (0–20 cm): evaporation-dominated with rapid seasonal fluctuations and weak moisture-salinity coupling (r = 0.07, n.s.); (2) Transition Zone (20–60 cm): dynamic equilibrium between upward and downward fluxes, intermediate correlation (r = 0.12, n.s.); (3) Subsurface Zone (60–100 cm): irrigation-driven redistribution with progressive seasonal accumulation, significant positive correlation (r = 0.25, p < 0.01), indicating downward redistribution culminating in deep accumulation (Aug–Sep) and late-season redistribution. Arrows indicate major fluxes: strong evapotranspiration (red upward), capillary rise (blue upward), and irrigation-driven downward leaching (green). The temporal dimension highlights July as the critical window when peak evapotranspiration drives maximum surface accumulation.

Implications for irrigation management

While these patterns are consistent with subsurface salt redistribution, definitive mechanistic proof would require multi-year monitoring and direct salt mass measurements. Despite the weak overall correlation (R^2 = 0.06), the

observed trend of increasing deep-layer salinity represents a risk signal that warrants attention. For land managers, ignoring this statistically significant but weak signal could lead to long-term hidden degradation. Our findings are not intended to establish precise predictive models, but rather to demonstrate the necessity of extending monitoring depth to 100 cm as a risk management practice. Even modest subsurface salt loading, if sustained over multiple seasons, could compromise long-term soil quality and irrigation effectiveness.

The seasonal EC redistribution patterns indicate the potential for progressive deep-layer accumulation, with corresponding changes in soil moisture profiles affecting salt transport mechanisms.

The observed subsurface accumulation pattern is consistent with potential vertical redistribution of salts, but does not constitute proof of long-term reservoir formation. Given the single-season design and EC-based estimation, the findings should be interpreted as relative dynamics supporting risk-oriented management rather than absolute salt mass assessment.

The application of EC-based estimation enabled rapid, non-destructive salinity assessment across all depths. Based on the observed seasonal patterns (Results, Fig 5), management strategies should prioritize early-season (April–May) high-volume leaching to prevent mid-season accumulation, while late-season applications should minimize deep percolation to reduce subsurface salt loading.

Since current practice merely relocates salts deeper rather than removing them, effective storage capacity may become increasingly constrained over time under continued subsurface accumulation. These dynamics address critical knowledge gaps in understanding complex salt transport dynamics across the vadose zone [24]. Similar subsurface accumulation patterns have been reported in the arid regions of Northwest China and the United States, indicating that deep-layer salt storage is a common yet underreported phenomenon in gravity irrigation systems [25,37]. Our findings suggest depth-differentiated approaches: surface desalination requires high-volume early-season leaching (April–May: 120–150 mm applications), while late-season applications should minimize deep percolation. The subsurface salt loading suggests that drainage infrastructure may play a critical role in supporting long-term sustainability—indicating that management attention could be focused on sites where subsurface EC exceeds 1000 μS/cm or shows progressive accumulation trends.

Based on these depth-dependent dynamics, Fig 7 presents a practical management framework for optimizing irrigation strategies.

Fig 7. Conceptual framework for adaptive irrigation management based on soil water-salt monitoring. The strategy relies on monthly monitoring at three depths (0–20, 40–60, 80–100 cm) to guide sequential decisions: (1) Initial Assessment (Apr–May): Surface EC >800 μS/cm triggers heavy leaching (120–

150 mm) targeting <400 μS/cm; lower values indicate standard irrigation (60–80 mm). (2) Subsurface Monitoring (Ongoing): Deep EC growth rate >10% signals accumulation risk, warranting irrigation reduction and drainage assessment. (3) Mid-season (Jun–Jul): Surface EC >800 μS/cm triggers supplemental leaching (40–60 mm); EC <400 μS/cm supports continued normal irrigation. (4) Late-season (Aug–Sep): Irrigation meets crop demand (40–60 mm) while avoiding deep percolation. (5) Critical Threshold: Ongoing monitoring triggers engineering intervention (drainage infrastructure) when deep accumulation reaches critical levels. Color coding denotes intensity: blue (monitoring & decisions), green (normal operations), yellow (adjusted strategy), and red (warning & intervention). The framework emphasizes continuous feedback and adaptive management. Note: Thresholds are conceptual guidance requiring local adaptation.

It should be noted that the threshold values and management responses illustrated in Fig 7 are intended as conceptual guidance based on observed patterns rather than prescriptive criteria, and should be adapted to specific local soil, crop, and hydrological conditions.

Limitations and future research

Our decision to estimate salt content from EC measurements rather than conduct gravimetric analysis on all 450 samples reflects a pragmatic balance between sampling density and laboratory throughput. The application of linear

conversion equations is a standard practice in salinity assessment [28]. However, readers should interpret the reported salt values as EC-derived estimates that capture relative salinity patterns rather than absolute salt masses. While the use of a fixed regional conversion factor introduces inherent uncertainty compared to direct weighing, it enables the comprehensive spatiotemporal assessment required to capture dynamic salt redistribution. This approach does not compromise our main conclusions regarding depth-dependent dynamics and seasonal salt redistribution patterns, as these are based on relative changes and EC-moisture coupling rather than absolute mass budgets. Importantly, our sampling design was intended for gradient analysis and process understanding rather than regional average estimation. Therefore, results are most applicable for risk identification and mechanism exploration, and should not be used to calculate regional-scale salt budgets without additional stratified sampling.

Monthly sampling may miss short-term dynamics following irrigation events, though it reflects time-averaged conditions experienced by crops. The absence of May data, due to continuous field irrigation that prevented site access, represents a limitation as May typically corresponds to peak irrigation period. While this data gap may have limited our ability to fully capture the immediate post-irrigation leaching response, the remaining five sampling months (April, June, July, August, September) still provide comprehensive

coverage of pre-irrigation, irrigation, and post-irrigation periods across the growing season. Our EC measurements in 1:5 extracts provide relative indices rather than in situ conditions. We did not measure individual ion concentrations, precluding assessment of sodium hazards. Most critically, this single-season study cannot definitively assess long-term trends—multi-year observations (5–10 years) with independent gravimetric validation are essential to determine whether subsurface salt loading represents progressive saturation or seasonal redistribution.

Future research should prioritize: (1) long-term monitoring to reveal time-to-saturation for salt reservoir capacity; (2) process-based modeling (e.g., HYDRUS, SWAP) to optimize irrigation schedules; (3) coupling field monitoring with emerging remote sensing and machine learning technologies [37-40] to upscale these point-based findings to the district level for precision salinity control, as successfully demonstrated in other large river deltas [41,42]; (4) ion-specific analysis to assess sodicity hazards; and (5) drainage pilot studies to evaluate effectiveness and optimize design parameters.

Conclusions

This study provides observational evidence of depth-dependent soil water-salt relationships in one of China's largest irrigation districts. Our monitoring of EC-derived salinity index indicates patterns consistent with potential subsurface salt redistribution that may pose challenges for the long-term

management of irrigated agriculture. Statistically significant but weak positive moisture-salinity correlations in subsurface layers (60–100 cm, $r = 0.25$; small effect size) and observed seasonal EC increases (42%) are consistent with the hypothesis of salt redistribution during irrigation. Using EC as a robust proxy enabled rapid field assessment across all depths, with analyses incorporating EC, moisture, and depth revealing strong depth-dependent modulation of salinity signals.

The results indicate that sustainable salinity control could benefit from moving from surface-focused leaching to integrated depth-differentiated strategies that consider enhanced drainage infrastructure. Based on the observed seasonal patterns, irrigation timing and volumes should be optimized to balance surface desalination with minimizing subsurface accumulation. Late-season subsurface EC patterns are consistent with vertical salt redistribution. Management attention might therefore be focused on evaluating subsurface drainage needs in areas where EC exceeds 1000 μS/cm.

These findings highlight limitations of conventional leaching-based management approaches that primarily focus on surface salt removal while overlooking subsurface redistribution. The observed patterns in EC-derived salinity index provide field-based indications of how salts may be redistributed within the soil profile under large-scale irrigation, contributing to our understanding of salinization dynamics in the Hetao Irrigation District and

potentially in other arid irrigation systems with similar conditions. From a management perspective, the results underscore the importance of considering strategies that facilitate effective salt removal from the soil profile, rather than approaches that predominantly relocate salts to deeper layers over time.

References

1. Qadir M, Quillérou E, Nangia V, Murtaza G, Singh M, Thomas RJ, et al. Economics of salt-induced land degradation and restoration. Nat Resour Forum. 2014;38(4):282–295. doi:10.1111/1477-8947.12054

2. Corwin DL. Climate change impacts on soil salinity in agricultural areas. Eur J Soil Sci. 2021;72(2):842–862. doi:10.1111/ejss.13010

3. Hassani A, Azapagic A, Shokri N. Global predictions of primary soil salinization under changing climate in the 21st century. Nat Commun. 2021;12(1):6663. doi:10.1038/s41467-021-26907-3

4. Singh A. Soil salinization and waterlogging: A threat to environment and agricultural sustainability. Ecol Indic. 2021;127:107748. doi:10.1016/j.ecolind.2021.107748

5. Berhe AA, Hopmans JW, Six J. Soil salinization: Implications for climate change impacts and adaptation. In: Climate Change and Soil Interactions. Amsterdam: Elsevier; 2022. pp. 555–578.

6. Butcher K, Wick AF, DeSutter T, Chatterjee A, Harmon J. Soil salinity: A threat to global food security. Agron J. 2016;108(6):2189–2200. doi:10.2134/agronj2016.06.0368

7. Yang J, Yao R, Wang X, Xie W, Zhang X, Zhu W, et al. Soil salinization in China: Current status, driving forces, and comprehensive management strategies. Agronomy. 2022;12(10):2523. doi:10.3390/agronomy12102523

8. Cao Z, Zhu T, Cai X. Hydro-agro-economic optimization for irrigated farming in an arid region: The Hetao Irrigation District, Inner Mongolia. Agric Water Manag. 2023;277:108095. doi:10.1016/j.agwat.2022.108095

9. Chen X, Wang Y, Guo Z, Xu J, Zhou L, Qiu G. Optimizing irrigation and drainage by considering agricultural hydrological process in arid farmland with shallow groundwater. Agric Water Manag. 2020;229:105303. doi:10.1016/j.agwat.2019.105303

10. Rengasamy P. World salinization with emphasis on Australia. J Exp Bot. 2006;57(5):1017–1023. doi:10.1093/jxb/erj108

11. Shrivastava P, Kumar R. Soil salinity: A serious environmental issue and plant growth promoting bacteria as one of the tools for its alleviation. Saudi J Biol Sci. 2015;22(2):123–131. doi:10.1016/j.sjbs.2014.12.001

12. Hillel D. Introduction to Environmental Soil Physics. Amsterdam: Elsevier Academic Press; 2004.

13. Wu T, Wang Z, Zhang L. Advanced strategies for controlling soil salinization in irrigated regions. Adv Agron. 2021;167:193–269. doi:10.1016/bs.agron.2021.01.005

14. Chen W, Hou Z, Wu L, Liang Y, Wei C. Evaluating salinity distribution in soil irrigated with saline water in arid regions of northwest China. Agric Water Manag. 2010;97(12):2001–2008. doi:10.1016/j.agwat.2010.03.008

15. Zaman M, Shahid SA, Heng L. Guideline for Salinity Assessment, Mitigation and Adaptation Using Nuclear and Related Techniques. Cham: Springer; 2018.

16. Corwin DL, Lesch SM. Apparent soil electrical conductivity measurements in agriculture. Comput Electron Agric. 2005;46(1–3):11–43. doi:10.1016/j.compag.2004.10.005

17. Tian F, Wu J, Liu W. Salinity stress effects on transpiration based on thermal infrared remote sensing. Geoderma. 2020;357:113961. doi:10.1016/j.geoderma.2019.113961

18. Zhang H, Schroder JL, Fuhrman JK, Basta NT, Storm DE, Payton ME. Path and multiple regression analyses of phosphorus sorption capacity. Soil Sci Soc Am J. 2005;69(4):1146–1151. doi:10.2136/sssaj2004.0114

19. Ivushkin K, Bartholomeus H, Bregt AK, Pulatov A, Kempen B, de Sousa L. Global mapping of soil salinity change. Remote Sens Environ. 2019;231:111260. doi:10.1016/j.rse.2019.111260

20. Peng Z, Wang J, Liu F. Seasonal dynamics of soil salinity and its relationship with groundwater depth. Catena. 2022;210:105904. doi:10.1016/j.catena.2021.105904

21. Ren D, Xu X, Hao Y, Huang G. Modeling the effects of irrigation water salinity on soil salt accumulation. J Arid Land. 2019;11(3):355–368. doi:10.1007/s40333-019-0105-0

22. Rhoades JD, Kandiah A, Mashali AM. The use of saline waters for crop production. FAO Irrigation and Drainage Paper 48. Rome: FAO; 1992.

23. Tan JY, Li AQ, Lei GP. Salt transport and balance in Hetao Irrigation District. Water Sci Eng. 2020;13(2):143–150. doi:10.1016/j.wse.2020.06.005

24. Hopmans JW, Qureshi AS, Kisekka I, Munns R, Grattan SR, Rengasamy P, et al. Critical knowledge gaps and research priorities in global soil salinity. Adv Agron. 2021;169:1–191. doi:10.1016/bs.agron.2021.03.001

25. Wang H, Wang N, Li J, Zhao Y. Simulation experiment on the effect of saline reclaimed water recharge on soil water and salt migration in Xinjiang, China. Water. 2023;15(1):238. doi:10.3390/w15010238

26. Zhang Q, Fan B, Wang Y. Optimization of subsurface drainage design in Hetao. Agric Water Manag. 2020;238:106213. doi:10.1016/j.agwat.2020.106213

27. Yin Z, Zhang Y, Zhao X. Experimental study on the effects of underground pipe and drainage in Hetao. Environ Earth Sci. 2020;79(1):1–13. doi:10.1007/s12665-019-8762-x

28. Rhoades JD. Salinity: Electrical conductivity and total dissolved solids. In: Sparks DL, editor. Methods of Soil Analysis, Part 3: Chemical Methods. Madison, WI: Soil Science Society of America; 1996. pp. 417–435.

29. Tong W, Chen X, Wen X, Chen F, Zhang H, Chu Q. Applying a salinity response function and zoning saline land for three field crops: A case study in the Hetao Irrigation District, Inner Mongolia, China. J Integr Agric. 2015;14(1):178–189. doi:10.1016/S2095-3119(14)60761-7

30. Tian F, Miao Q, Shi H, Li R, Dou X, Duan J, et al. Study on water and salt transport under different subsurface pipe arrangement conditions in severe saline-alkali land in Hetao Irrigation District with DRAINMOD model. Water. 2023;15(16):3001. doi:10.3390/w15163001

31. Cohen J. Statistical Power Analysis for the Behavioral Sciences. 2nd ed. Hillsdale, NJ: Erlbaum; 1988.

32. Seabold S, Perktold J. Statsmodels: Econometric and statistical modeling with Python. Proceedings of the 9th Python in Science Conference. 2010:92–96. doi:10.25080/Majora-92bf1922-011

33. Hillel D. Environmental Soil Physics. San Diego: Academic Press; 1998.

34. Bresler E, McNeal BL, Carter DL. Saline and Sodic Soils: Principles-Dynamics-Modeling. Berlin: Springer-Verlag; 1982.

35. Minhas PS, Ramos TB, Ben-Gal A, Pereira LS. Coping with salinity in irrigated agriculture: Crop evapotranspiration and water management issues. Agric Water Manag. 2020;227:105832. doi:10.1016/j.agwat.2019.105832

36. Zhu Q, Li H, Zhang J, Zhou J. Spatiotemporal dynamics of soil salinization in the Yellow River Delta under hydrological connectivity. Catena. 2024;235:107643. doi:10.1016/j.catena.2023.107643

37. Scudiero E, Skaggs TH, Corwin DL. Regional scale soil salinity assessment using Landsat ETM+ canopy reflectance. Remote Sens Environ. 2017;169:279–292. doi:10.1016/j.rse.2015.08.026

38. Gorji T, Sertel E, Tanik A. Monitoring soil salinity via remote sensing technology under data scarce conditions: A review. Ecol Indic. 2017;74:284–296. doi:10.1016/j.ecolind.2016.11.017

39. Hu J, Peng J, Zhou Y, Xu D, Zhao R, Jiang Q, et al. Quantitative estimation of soil salinity using UAV-borne hyperspectral and satellite multispectral images. Remote Sens. 2019;11(7):736. doi:10.3390/rs11070736

40. Wang S, Chen Y, Wang M. Weakly supervised deep learning for segmentation of remote sensing imagery. Remote Sens. 2020;12(2):207. doi:10.3390/rs12020207

41. El-Naggar A, El-Naggar AH, Sewale BE, Zenebe A. Soil properties and spatial distribution of soil salinity in the Nile Delta. PLoS ONE. 2020;15(11):e0241772. doi:10.1371/journal.pone.0241772

42. Hoa PV, Giang NV. Mapping soil salinity in the Mekong Delta using Sentinel-2 imagery and machine learning. PLoS ONE. 2023;18(3):e0283690. doi:10.1371/journal.pone.0283690

Supporting Information

S1 Dataset. Raw data. The complete dataset of soil moisture, EC, and salt content used in this study. (CSV)

Click here to access/download;Figure;Figure_1.tif

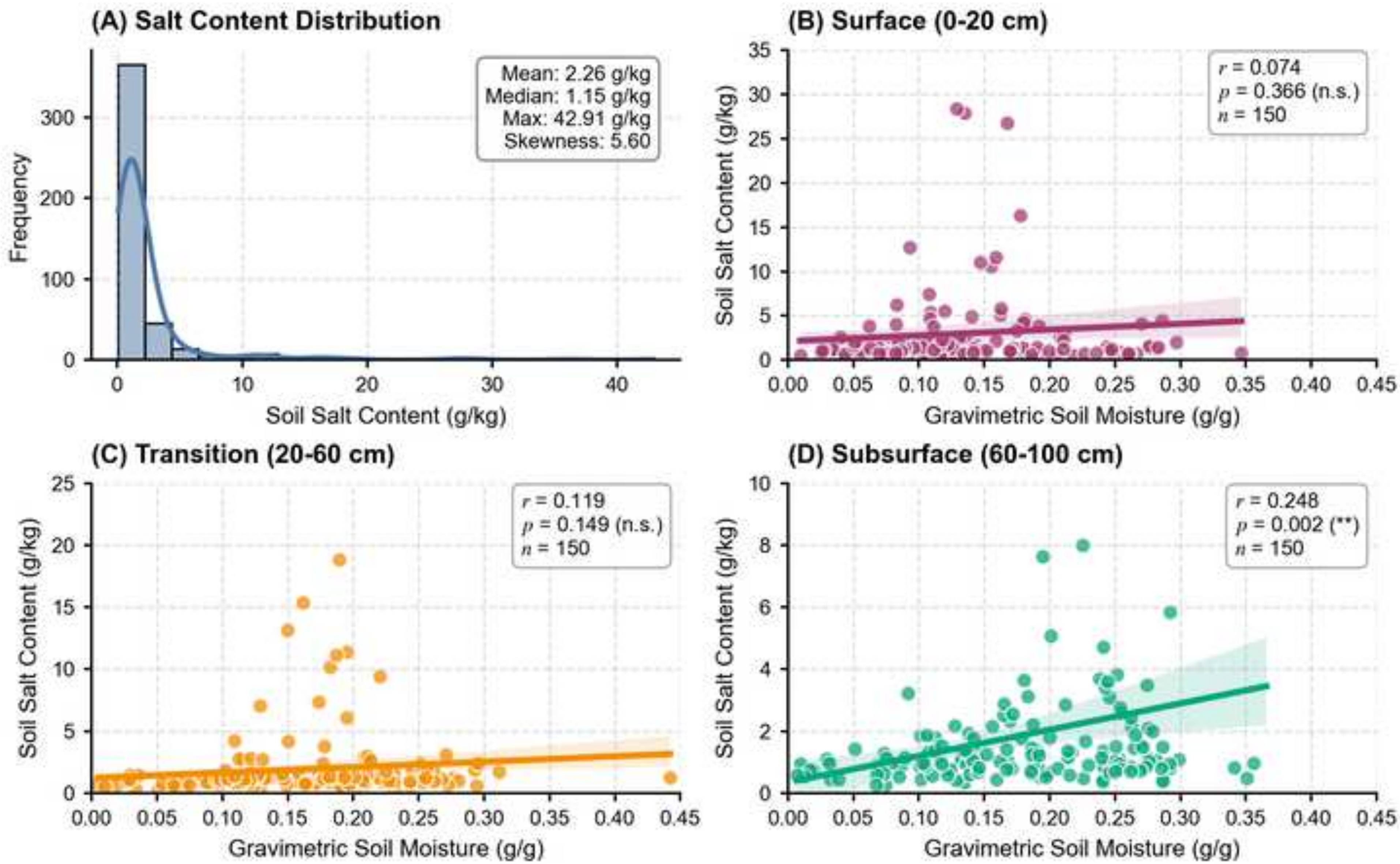

Click here to access/download;Figure;Figure_2.tif

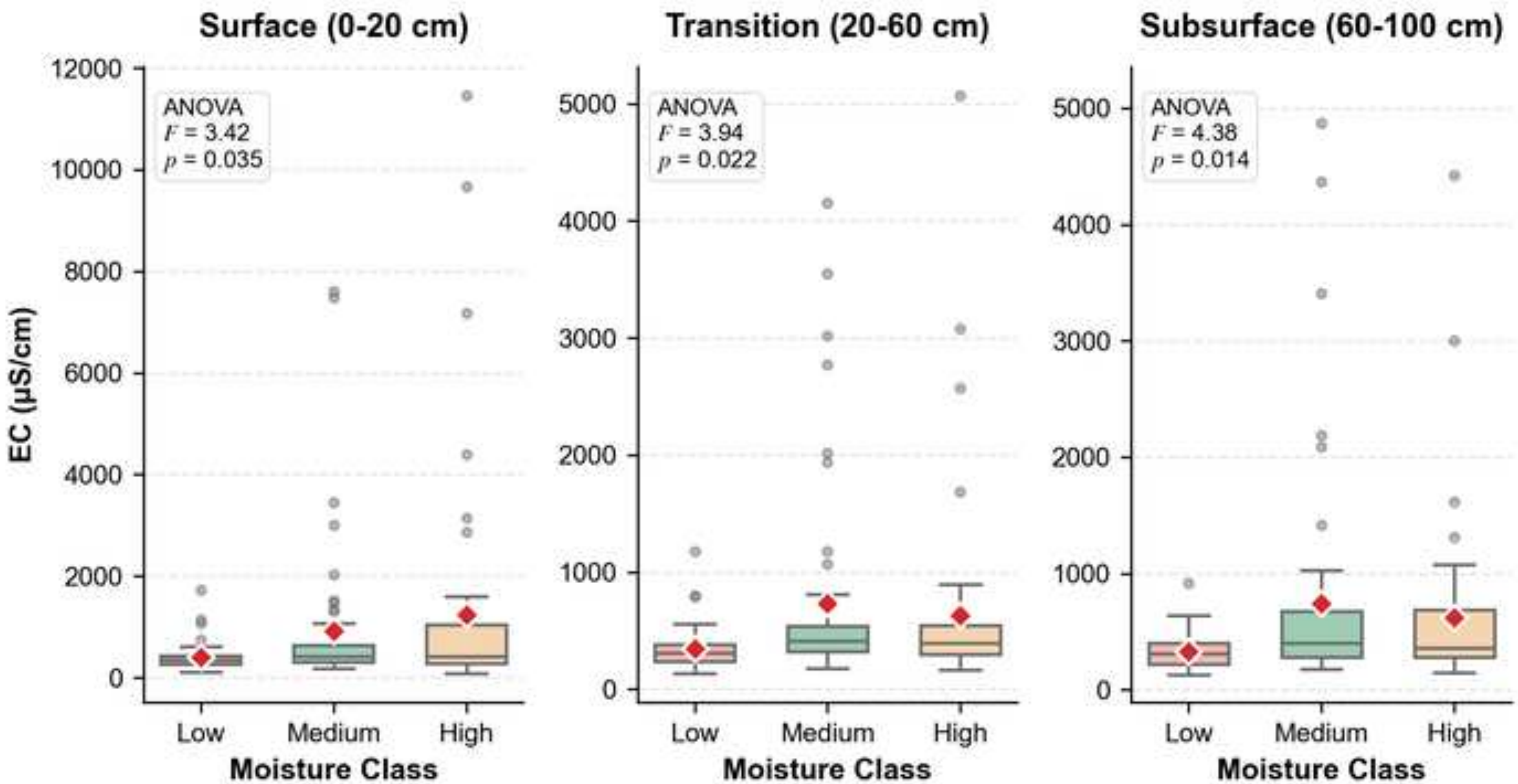

Click here to access/download;Figure;Figure_3.tif

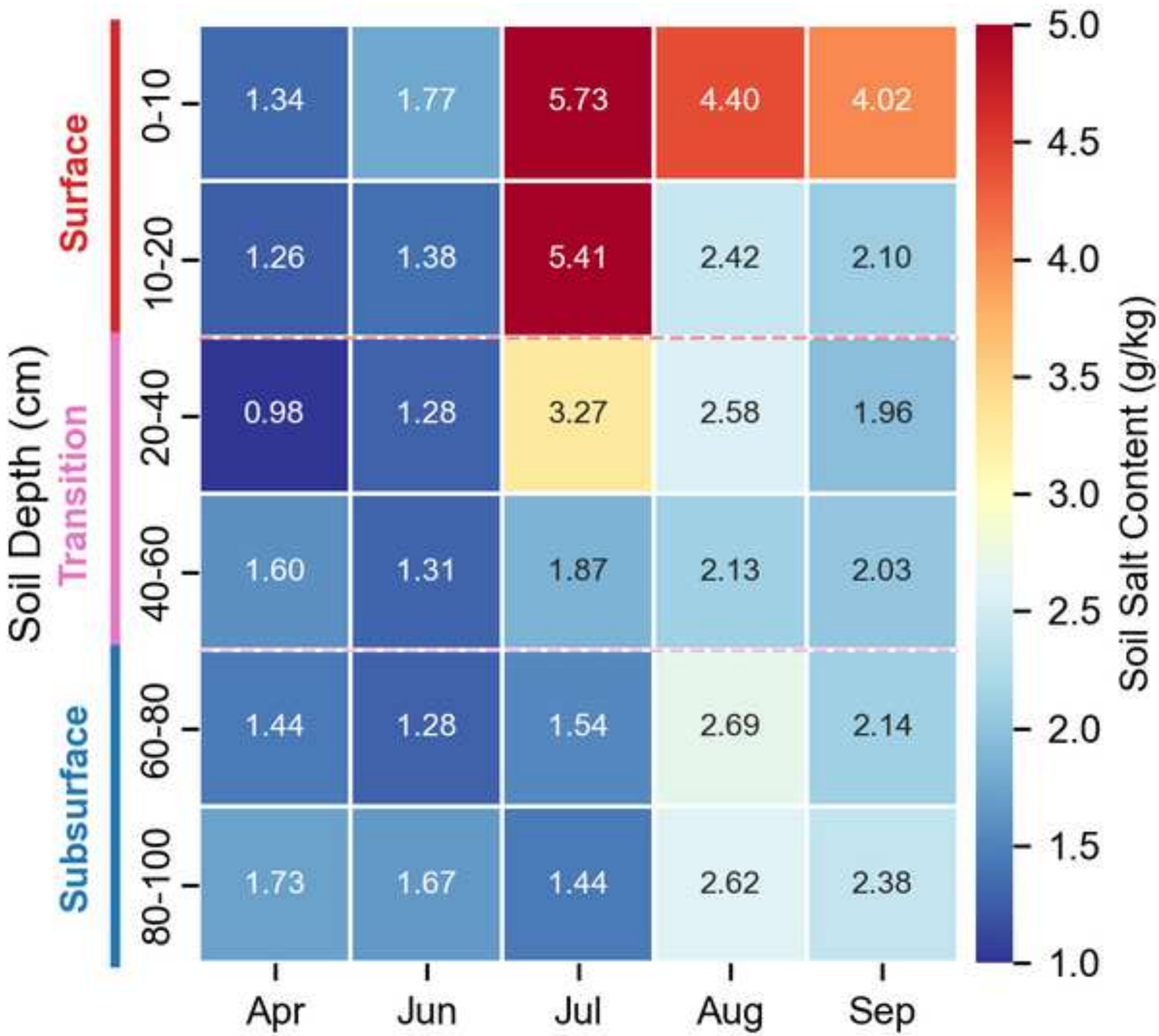

Click here to access/download;Figure;Figure_4.tif

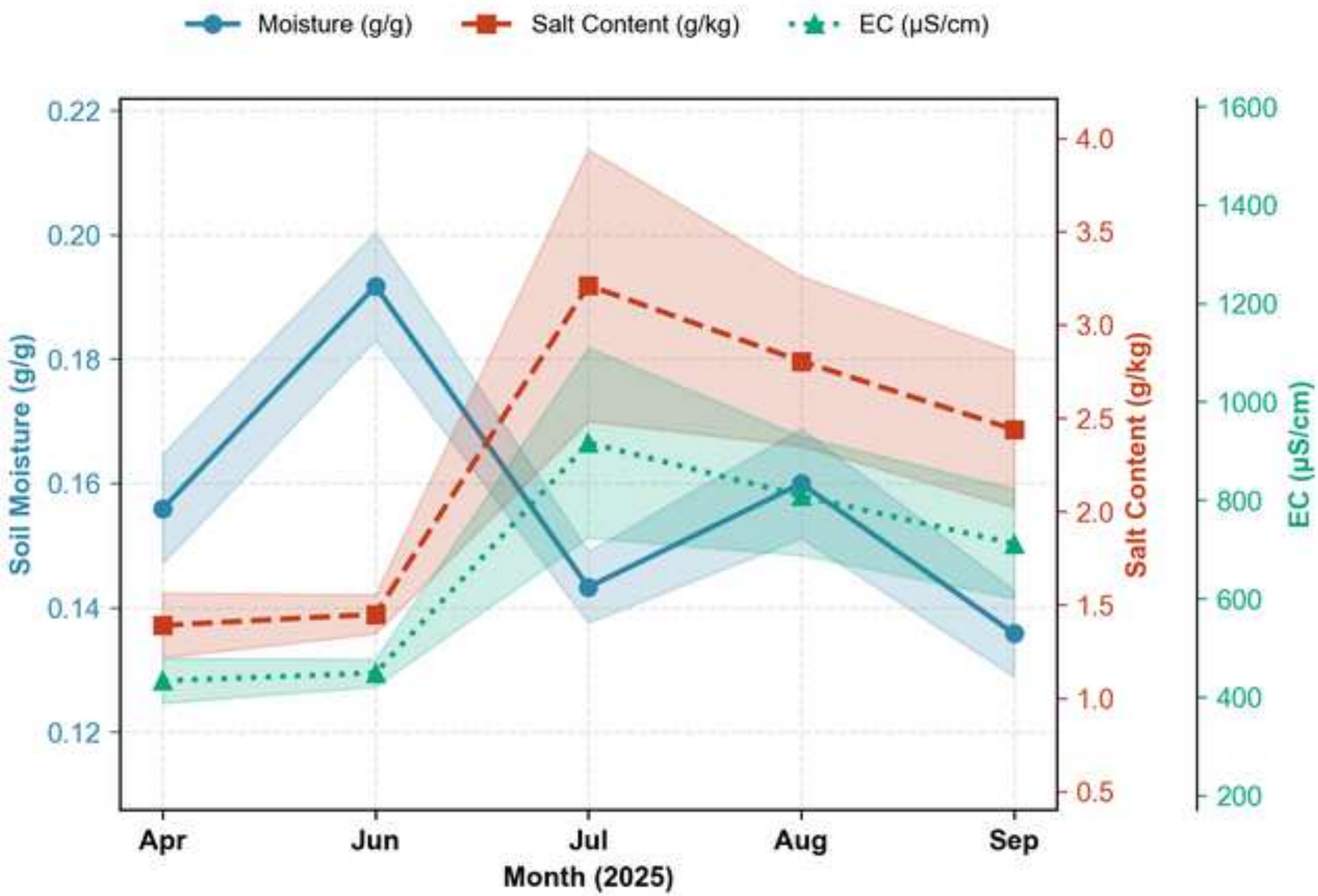

Click here to access/download;Figure;Figure_5.tif

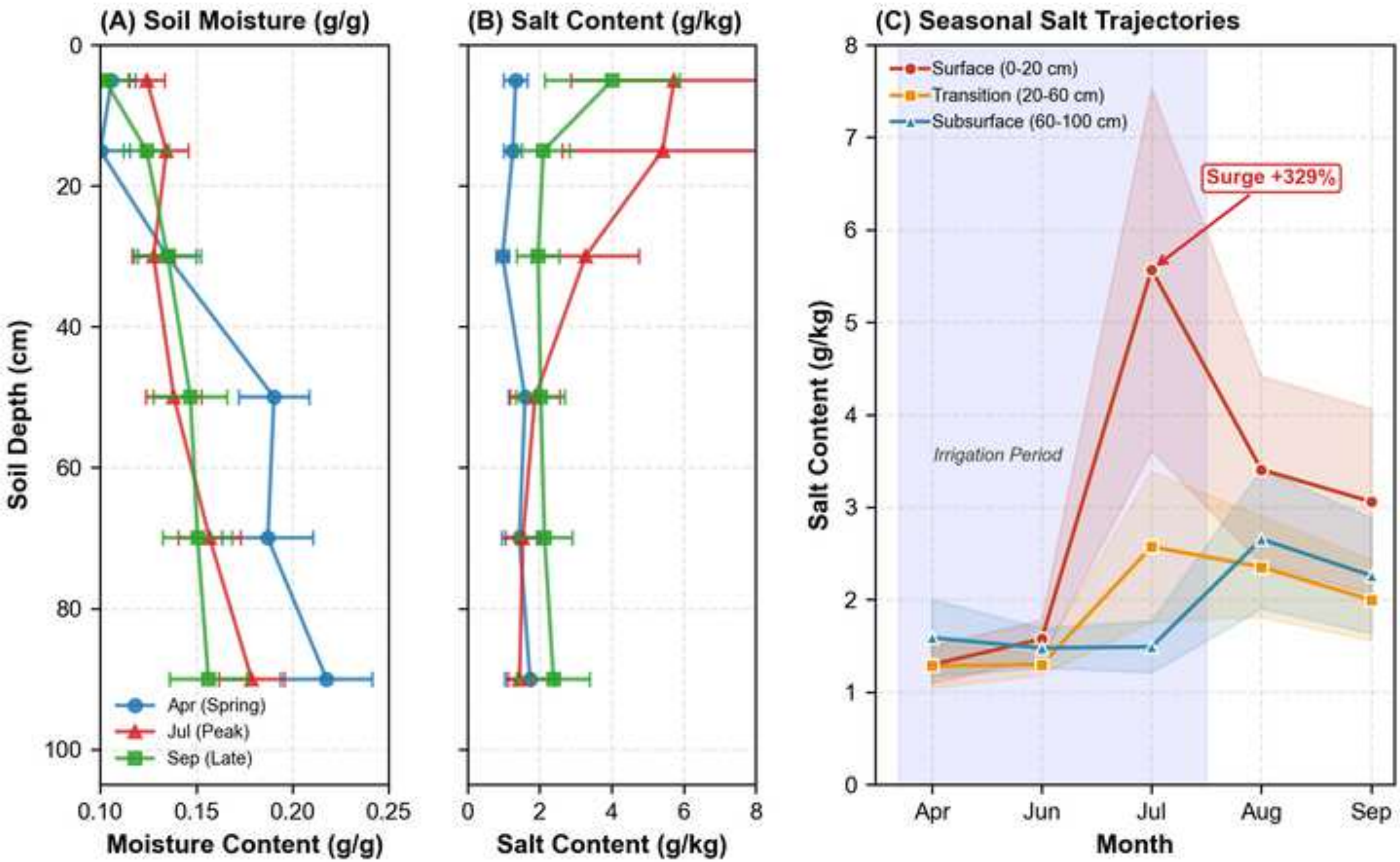

Click here to access/download;Figure;Figure_6.tif

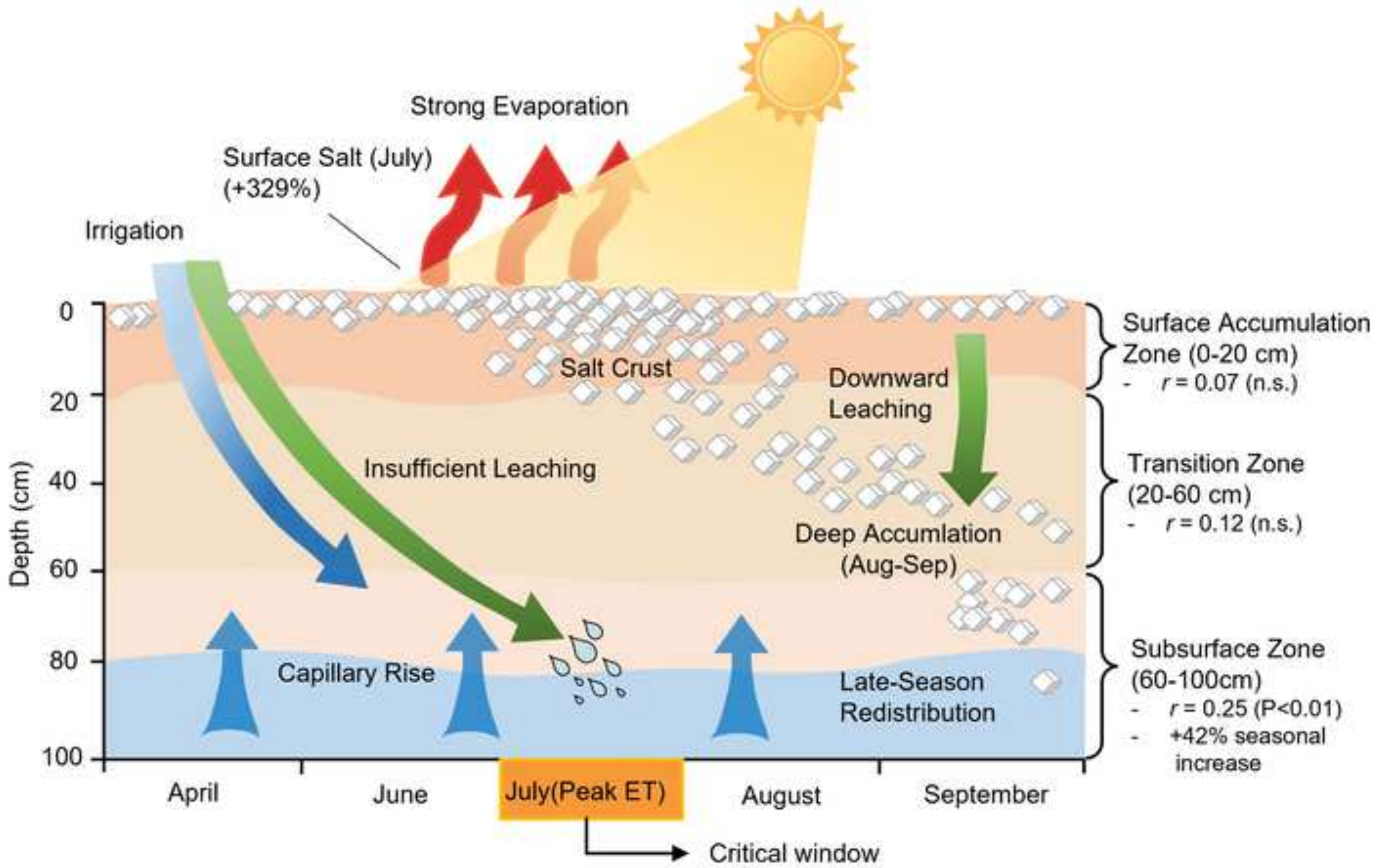

Click here to access/download;Figure;Figure_7.tif

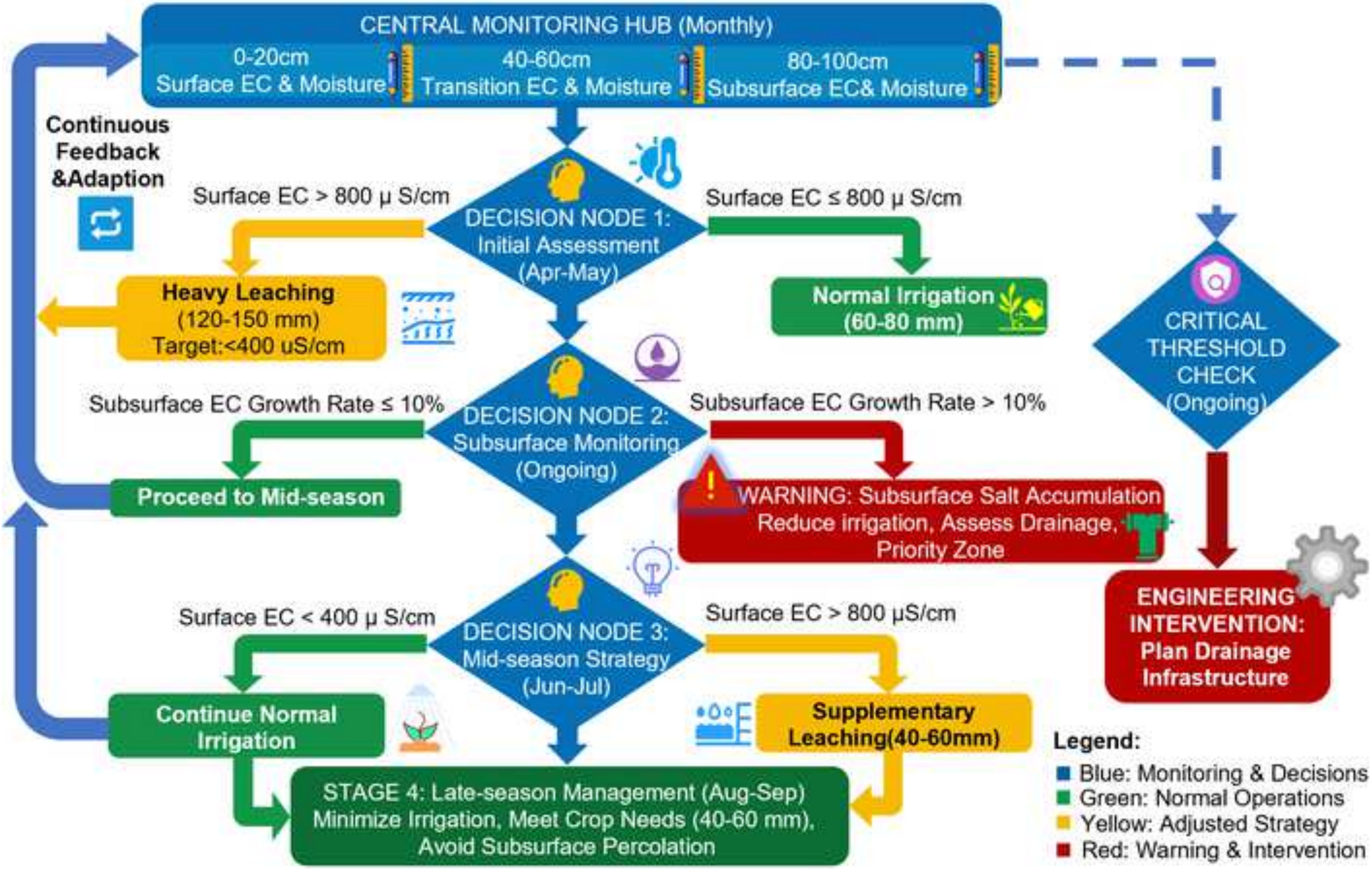

Click here to access/download
Supporting Information
S1_Dataset.csv
