# Supplementary material for: Depth-dependent soil water-salt relationships in the Hetao Irrigation District, arid Northwest China: Implications for sustainable management
Source: PLoS One. 2026 Oct 20;21(10):e0360976. doi: 10.1371/journal.pone.0360976 (PMC13682659; doi:10.1371/journal.pone.0360976)
Supplement: Supplementary file 2 [file pone.0360976.s002.docx]

The manuscript addresses an important topic and contains valuable field data. However, substantial revisions are required before it can be considered for publication. The primary concerns relate to methodological limitations, overinterpretation of weak statistical relationships, formatting and language issues, and insufficient clarification of novelty. The following points are given below-

**1.** The manuscript claims to provide new insights into depth-dependent salinity dynamics. However, similar studies have already reported subsurface salt accumulation in arid irrigation systems. The authors should more clearly articulate what is novel in their work (e.g., scale of dataset, analytical framework, or methodological approach).

**2.** The reported correlation between soil moisture and salinity in deep layers (r = 0.25, R² = 0.06) is statistically significant but weak. The manuscript repeatedly interprets this relationship as evidence of meaningful coupling and redistribution processes. This interpretation appears overstated given the low explanatory power. The authors should clearly distinguish between statistical significance and practical significance

**3.** Salt content is calculated directly from electrical conductivity (EC), yet EC is subsequently used in regression analyses and to interpret salinity dynamics. This introduces an inherent circularity in the analytical framework. Although this limitation is acknowledged, it should be more explicitly discussed and accompanied by a more cautious interpretation of regression results and derived relationships.

**4.** The study is based on observations from a single growing season (2025), which limits its ability to support conclusions regarding long-term salinity dynamics or sustainability. Consequently, statements referring to “long-term accumulation” and “sustainability risks” are not fully substantiated by the dataset. The authors should revise these claims to reflect that the findings represent short-term observational evidence rather than long-term trends.

**5.** The absence of sampling in May, due to irrigation constraints, significant limitation, as this period likely coincides with peak leaching activity. The manuscript should more critically assess the implications of this data gap and avoid drawing strong conclusions about leaching dynamics in the absence of observations from this key period.

**6.** The stratified sampling approach deliberately includes sites across a range of salinity gradients to capture variability. While this design is appropriate for process-oriented analysis, it limits the representativeness of the results at the regional scale. The authors should clearly state that the findings are not intended for regional extrapolation and should be interpreted as indicative of patterns rather than representative averages.

**7.** The study relies exclusively on electrical conductivity (EC)-based estimates to infer soil salinity, without incorporating direct gravimetric measurements or ion-specific analyses (e.g., Na⁺, Cl⁻). This reliance constrains the accuracy of absolute salt quantification and prevents assessment of sodicity-related risks. This limitation should be more explicitly acknowledged and emphasized in both the Methods and Discussion sections.

**8.** Key findings, particularly those related to subsurface accumulation and weak correlations, are reiterated across multiple sections. The manuscript would benefit from improved conciseness by minimizing repetition.

**9.** All figures and tables should be clearly referenced and adequately described within the text. Captions should be revised to improve clarity and conciseness, avoiding unnecessary repetition of information already presented in the main text.

**Overall, the study has potential but requires careful revision before it is suitable for publication.**
