# Supplementary material for: Depth-dependent soil water-salt relationships in the Hetao Irrigation District, arid Northwest China: Implications for sustainable management
Source: PLoS One. 2026 Oct 20;21(10):e0360976. doi: 10.1371/journal.pone.0360976 (PMC13682659; doi:10.1371/journal.pone.0360976)
Supplement: Supplementary file 4 [file pone.0360976.s006.docx]

**Response to Reviewers**

*Manuscript:* *Depth-dependent soil water-salt relationships in the Hetao Irrigation District, arid Northwest China: Implications for sustainable management*

Dear Editor and Reviewers,

We thank the reviewers for their careful reading and constructive comments. The feedback has been valuable in identifying areas for improvement and aspects requiring further clarification in the original submission. We have revised the manuscript in response to all nine points, and the manuscript has been strengthened accordingly.

All changes are shown in the track-changes version of the manuscript. Key revised passages are reproduced as indented blocks below, with page and line numbers referring to the Revised Manuscript (34 pp.). Deleted content is noted without line numbers, as those passages no longer appear in the revised manuscript.

**Point 1. Insufficient articulation of novelty**

*The manuscript claims to provide new insights into depth-dependent salinity dynamics. However, similar studies have already reported subsurface salt accumulation in arid irrigation systems. The authors should more clearly articulate what is novel in their work (e.g., scale of dataset, analytical framework, or methodological approach).*

We agree that the original manuscript did not adequately distinguish our contribution from prior work. The following revisions address this.

***1-a. Abstract — comparative closing sentence***

The Abstract now closes with a sentence that identifies our dataset and analytical framework as key differentiators (Revised MS, p.2 Line 37 – p.3 Line 40):

*Compared with previous studies, this work provides a depth-resolved, multi-site observational dataset combined with a stratified analytical approach, offering additional evidence for interpreting soil salinity dynamics under heterogeneous field conditions.*

***1-b. Introduction — novelty paragraph***

The Introduction has been rewritten to position our study against the literature and articulate three methodological distinctions (Revised MS, p.4 Line 74 – p.5 Line 88):

*While subsurface salt accumulation and depth-dependent salinity dynamics have been reported in previous studies, existing research is often based on limited sampling depth, single-site observations, or simplified assumptions of soil profile uniformity.*

*This study differs from previous work in three main aspects. First, it is based on a multi-site field dataset (15 locations) covering the full soil profile (0–100 cm) across a wide salinity gradient. Second, it applies a depth-stratified analytical framework that combines correlation analysis with moisture-class group comparison to better capture patterns under heterogeneous field conditions. Third, the study adopts a pattern-oriented interpretation, focusing on identifying consistent signals in observational data rather than establishing strong predictive or mechanistic relationships.*

*These aspects allow a more comprehensive examination of depth-dependent water–salt relationships and provide additional evidence for understanding salinity redistribution processes in arid irrigation systems.*

***1-c. Introduction — literature attribution***

A sentence was added noting that deep-layer accumulation “has been reported to potentially influence long-term sustainability in previous studies” (Revised MS, p. 3, Lines 54–59), attributing prior findings while positioning our work as an extension.

***1-d. Conclusions — novelty statement***

A new paragraph was added to the Conclusions (Revised MS, p. 27, Lines 539–544):

*The novelty of this study lies in integrating multi-site full-profile (0–100 cm) observations with a depth-stratified analytical framework under large-scale irrigation conditions. This approach helps reveal subtle depth-dependent signals across the soil profile, although these findings must be interpreted with caution regarding long-term salinity dynamics.*

***1-e. Methods — comparison with conventional approaches***

A sentence comparing our group-comparison strategy with conventional regression-based approaches was added (Revised MS, p. 10, Line 204 – p. 11, Line 213).

**Point 2. Overinterpretation of a weak statistical relationship (r = 0.25, R² = 0.06)**

*The reported correlation between soil moisture and salinity in deep layers (r = 0.25, R² = 0.06) is statistically significant but weak. The manuscript repeatedly interprets this relationship as evidence of meaningful coupling and redistribution processes. This interpretation appears overstated given the low explanatory power. The authors should clearly distinguish between statistical significance and practical significance.*

This concern has been addressed throughout all sections, as detailed below.

***2-a. Abstract***

The phrase “small effect size” has been removed and replaced with an explicit statement of limited explanatory power (Revised MS, p. 2, Lines 31–33):

*(r = 0.25, p < 0.01, R² = 0.06), indicating limited explanatory power. This pattern is consistent with a subtle tendency of downward salt redistribution.*

The Abstract’s sentence referring to “the long-term effectiveness of leaching-based salinity management” and the closing application sentence were deleted.

***2-b. Results — correlation reporting***

The deep-layer correlation paragraph now foregrounds the practical interpretation of R² (Revised MS, p. 14, Line 282 – p. 15, Line 288):

*Critically, the deep subsurface layers (60–100 cm) showed a statistically significant but weak positive correlation (r = 0.25, P < 0.01). However, the low coefficient of determination (R² = 0.06) indicates limited practical significance, with soil moisture explaining only a small fraction of the variability in deep-layer salinity. This suggests that while a statistical association exists, moisture alone is insufficient to characterize salinity dynamics in these layers.*

***2-c. Results — group-comparison paragraph***

Causal language has been replaced by explicit signal-pattern framing (Revised MS, p. 16, Lines 304–307):

*This group-level distinction indicates that EC signals differ across moisture classes at all depths. However, because the derived salinity index is a linear transformation of EC, these group comparisons should be interpreted as patterns in the ionic signal rather than evidence of independently confirmed moisture-driven salt redistribution.*

***2-d. Results — seasonal deep-layer trend***

The phrase “long-term threat” and causal attribution to irrigation leaching have been replaced (Revised MS, p. 19, Lines 376–381):

*The seasonal increase of 42% in deep layers from April to September indicates a net seasonal redistribution of EC-derived salinity within the soil profile. This subsurface accumulation may indicate early subsurface salt storage, with salts at 60–100 cm depth potentially available for upward transport during dry periods, although its longer-term implications require multi-season validation.*

***2-e. Discussion — subsection heading and framing***

The subsection heading was changed from “Depth-dependent water-salt coupling” to “Depth-dependent water-salt associations.” Instances of “genuine solute transport” were replaced by “possible solute transport,” and certainty statements now carry explicit hedges referencing R² and the absence of May observations. Two representative passages (Revised MS, p. 21, Lines 409–418 and p. 22, Lines 429–432):

*This depth-stratified signal pattern suggests that the deep-layer moisture–salinity association may reflect possible solute transport processes; however, this interpretation remains tentative given the low explanatory power (R² = 0.06) and the observational nature of the dataset, as well as the absence of observations during the peak irrigation period in May. The absence of correlation in surface layers — where moisture effects on EC readings would be most pronounced due to higher variability — further supports the interpretation that deeper correlations represent real redistribution of the EC signal rather than measurement artifacts.*

*Overall, these depth-dependent associations should be interpreted as indicative patterns rather than evidence of strong mechanistic coupling, particularly given the limited explanatory power of the statistical relationships.*

***2-f. Discussion and Conclusions***

Language such as “could lead to long-term hidden degradation,” “More concerning,” and “are consistent with vertical salt redistribution” has been moderated throughout. The Conclusions now open with a statement conditioning causal inference on the short-term study design, and “small effect size” has been removed from the Conclusions as well. Three further terminology substitutions were applied to eliminate residual “coupling” and “decoupling” language that implied mechanistic coupling inconsistent with the weak statistical evidence: (i) Abstract (Revised MS, p. 2, Line 25): “depth-dependent decoupling of water–salt dynamics” replaced by “depth-dependent divergence in water–salt associations”; (ii) Results (Revised MS, p. 14, Line 274): “moisture-salinity coupling patterns” replaced by “moisture–salinity association patterns”; (iii) Results (Revised MS, p. 14, Line 280): “the moisture-salinity relationship shifted” shortened to “the relationship shifted,” removing the redundant attributive phrase.

**Point 3. Circularity in the analytical framework (EC → salinity index → EC regression)**

*Salt content is calculated directly from electrical conductivity (EC), yet EC is subsequently used in regression analyses and to interpret salinity dynamics. This introduces an inherent circularity in the analytical framework. Although this limitation is acknowledged, it should be more explicitly discussed and accompanied by a more cautious interpretation of regression results and derived relationships.*

We fully accept this critique. Revisions address the issue at the terminological, methodological, and interpretive levels.

***3-a. Global terminology replacement***

The variable formerly called “Soil Salt Content (SSC)” is now named “EC-derived salinity index (derived salinity)” throughout the manuscript — text, figures, tables, and captions. It is introduced in the Methods (Revised MS, p. 8, Lines 160–163):

*An EC-derived salinity index (hereafter referred to as derived salinity) was estimated from EC1:5 measurements using a published conversion equation calibrated for Hetao Irrigation District soils [29]: Derived Salinity (g/kg) = 0.0037657 × EC1:5 (μS/cm) − 0.2405 (Eq. 1)*

This renaming is applied consistently to Table 1, Table 2, and Table 3 headers, and throughout Results and Discussion text.

***3-b. Regression framework respecification***

The multiple regression model no longer treats EC and the derived salinity index as simultaneous predictors. Moisture and depth are the independent variables (Revised MS, p. 10, Lines 195–198):

*Multiple linear regression and correlation analyses were used to examine how independent explanatory variables (moisture and depth) relate to the EC-derived salinity index. Model performance used R² and root mean square error (RMSE).*

***3-c. Interpretive framing — Methods***

A new paragraph explains the analytical approach governing all statistical results (Revised MS, p. 12, Lines 230–234):

*To address the inherent circularity, we emphasize that our statistical models do not treat EC and derived salinity as independent variables. Rather, regressions evaluate how environmental factors interact with the EC signal. Consequently, all results are interpreted as spatial patterns of soluble ions rather than predictive mechanistic models.*

***3-d. Variable usage rules — start of Results***

A clarification paragraph at the opening of Results states the usage rules explicitly (Revised MS, p. 13, Lines 253–257):

*The EC-derived salinity index is used as the primary salinity variable in all analyses. EC1:5 values are reported only where direct comparison with existing literature requires conductivity units. Because these two metrics are linear transformations of the same measurement, they are not used simultaneously as independent predictors in statistical models.*

***3-e. Limitations section***

The Limitations section has been reorganised to make the mathematical relationship between EC and the derived salinity index explicit and to acknowledge its consequences for interpretation (Revised MS, p. 25 Line 505 – p. 26, Line 512). See also Point 7.

**Point 4. Single-season data cannot support long-term conclusions**

*The study is based on observations from a single growing season (2025), which limits its ability to support conclusions regarding long-term salinity dynamics or sustainability. Statements referring to ‘long-term accumulation’ and ‘sustainability risks’ are not fully substantiated. The authors should revise these claims to reflect short-term observational evidence rather than long-term trends.*

Every passage implying multi-year validity has been revised, spanning Abstract through Conclusions.

***4-a. Abstract***

The sentence referring to “the long-term effectiveness of leaching-based salinity management” and the closing application sentence were deleted (see also Point 2-a).

***4-b. Results — seasonal deep-layer paragraph***

“Long-term threat” now reads “may indicate early subsurface salt storage … although its longer-term implications require multi-season validation” (full text quoted under Point 2-d, Revised MS, p. 19, Lines 376–381).

***4-c. Discussion — subsurface accumulation passage***

The passage describing a “potential risk that continued accumulation could pose long-term challenges” now reads (Revised MS, p. 21, Line 419– – p. 22, Line 429):

*The observed subsurface accumulation pattern (Results, Fig 3) aligns with reported deep-layer salt storage in arid irrigation systems [25,37], and is consistent with potential redistribution patterns; however, these interpretations remain speculative given the weak correlation strength and the low coefficient of determination (R² = 0.06), highlighting the need for multi-season validation … A potential concern is that these stored salts may return to the rooting zone under dry conditions, as suggested in previous studies [37], although this process warrants further investigation beyond the current single-season dataset.*

***4-d. Limitations and Conclusions***

The Limitations section now treats the temporal constraint as a “critical data gap” (Revised MS, p. 26, Lines 513–522; full text under Point 5-b). The Conclusions open with the acknowledgment that “causal inference is constrained by the short-term study design and the absence of May observations” (Revised MS, p. 27, Lines 538–539).

**Point 5. Absence of May data and implications for leaching interpretation**

*The absence of sampling in May, due to irrigation constraints, is a significant limitation, as this period likely coincides with peak leaching activity. The manuscript should more critically assess the implications of this data gap and avoid drawing strong conclusions about leaching dynamics in the absence of observations from this key period.*

The May data gap was mentioned briefly in the original but not treated as a major interpretive constraint. It is now foregrounded in three locations.

***5-a. Results — seasonal dynamics opening***

A new paragraph at the opening of the Seasonal dynamics subsection establishes the data gap as the controlling interpretive frame (Revised MS, p. 17, Lines 337–343):

*Salt redistribution processes operate at different timescales across soil depths. Because monthly sampling was conducted without observations in May — the period most likely corresponding to peak leaching activity — the dataset captures net seasonal changes rather than the redistribution dynamics themselves. Consequently, conclusions about leaching effectiveness, peak salt movement, and irrigation-driven redistribution pathways are not directly supported by the data and should be treated as inferential.*

***5-b. Limitations — upgraded to “critical data gap”***

In the Limitations section, the May gap has been elevated from a parenthetical note to a “critical data gap” (Revised MS, p. 26, Lines 513–522):

*Temporally, the reliance on a single growing season restricts the findings to short-term observational evidence. Monthly sampling may miss rapid responses following irrigation events, and the absence of May data — due to continuous irrigation preventing site access — represents a critical data gap during the presumed peak leaching period. Consequently, interpretations of irrigation-driven processes remain inferential. Ultimately, the combination of missing peak-period observations and the lack of multi-season data limits our ability to assess long-term sustainability, highlighting the need for multi-year monitoring to independently validate these depth-dependent redistribution trends.*

***5-c. Future Research***

Future Research item (1) now explicitly calls for “high-frequency monitoring during peak irrigation events to capture transient leaching dynamics” (Revised MS, p. 26, Lines 523–524), directly translating the recognised gap into a concrete research direction.

**Point 6. Regional representativeness of the stratified sampling design**

*The stratified sampling approach deliberately includes sites across a range of salinity gradients to capture variability. While this design is appropriate for process-oriented analysis, it limits the representativeness of the results at the regional scale. The authors should clearly state that the findings are not intended for regional extrapolation and should be interpreted as indicative of patterns rather than representative averages.*

***6-a. Results — scope caveat***

A caveat at the opening of Results ensures readers encounter the limitation before any data are presented (Revised MS, p. 12, Lines 237–239):

*All salinity values reported in this section are EC-derived estimates (Eq. 1) and are intended to characterize variability across the sampled salinity gradient rather than represent regional averages.*

***6-b. Limitations — regional extrapolation***

The Limitations section now explicitly states that the sampling design “deliberately targeted salinity gradients for process-oriented interpretation; therefore, these results should not be interpreted as regional averages or used to estimate large-scale salt budgets without additional stratified sampling” (Revised MS, p. 26, Lines 509–512).

***6-c. Introduction — analytical framing***

The rewritten Introduction novelty paragraph (Revised MS, p.4 Line 74 – p.5 Line 88; full text under Point 1-b) describes the study as adopting “a pattern-oriented interpretation, focusing on identifying consistent signals in observational data rather than establishing strong predictive or mechanistic relationships,” further contextualising the scope.

**Point 7. EC-only approach: absence of gravimetric and ion-specific data**

*The study relies exclusively on electrical conductivity (EC)-based estimates to infer soil salinity, without incorporating direct gravimetric measurements or ion-specific analyses (e.g., Na⁺, Cl⁻). This reliance constrains the accuracy of absolute salt quantification and prevents assessment of sodicity-related risks. This limitation should be more explicitly acknowledged and emphasized in both the Methods and Discussion sections.*

Two dedicated passages — one in Methods and one in Discussion — now address this concern directly.

***7-a. Methods — methodological constraints***

Revised text (Revised MS, p. 9, Lines 172–181):

*Methodological limitations of the EC-based approach. Because no direct gravimetric quantification or ion-specific analyses were conducted, three constraints apply to this methodology. First, the estimated values represent a relative index of salinity rather than precise in situ salt mass, carrying inherent regional conversion uncertainty. Second, the spatial and temporal variability of specific ionic compositions (e.g., SO₄²⁻, HCO₃⁻) cannot be resolved from EC alone. Third, sodicity-related hazards (e.g., sodium adsorption ratio, exchangeable sodium percentage) cannot be assessed. These limitations must be considered when interpreting quantitative results and management implications.*

***7-b. Discussion — sodicity threshold caveat***

Revised text (Revised MS, p. 24, Lines 481–486):

*It should be noted, however, that EC-based thresholds cannot indicate sodicity-related hazards (e.g., sodium adsorption ratio or exchangeable sodium percentage); elevated EC alone does not confirm alkalisation risk without ion-specific analysis. These thresholds are therefore proposed as indicators for prioritising further monitoring, not as criteria for salinity classification or remediation decisions.*

***7-c. Limitations section***

The Limitations section has been reorganised so that the EC-derived salinity index is first introduced as a mathematical transformation of EC, with the three constraints above reiterated. The phrase “risk-oriented management” has been replaced by “monitoring-based management” throughout the manuscript (e.g., Revised MS, p. 23, Line 460).

***7-d. Fig 6 — removal of statistical annotations***

All r-values, p-values, and percentage increases (+329%, +42%) have been removed from the Fig 6 panel. These values were derived from the EC signal and should not be presented as visual annotations without acknowledging their basis; their removal prevents misleading emphasis on results that cannot be verified against ion-specific data.

**Point 8. Repetition and conciseness**

*Key findings, particularly those related to subsurface accumulation and weak correlations, are reiterated across multiple sections. The manuscript would benefit from improved conciseness by minimizing repetition.*

The following passages were deleted in full:

• Methods: Redundant paragraph on EC conversion equation validation.

• Discussion: Detailed figure element descriptions now integrated into figure captions.

• Conclusions: Removed repetition of statistical results from Results section.

Together with the caption revisions described under Point 9, these deletions reduce the manuscript by one full page (from 35 to 34 pp.).

**Point 9. Figure and table captions: clarity, conciseness, and redundancy**

*All figures and tables should be clearly referenced and adequately described within the text. Captions should be revised to improve clarity and conciseness, avoiding unnecessary repetition of information already presented in the main text.*

All seven figures were revised. The approach throughout: retain only information needed for immediate interpretation of the visual (axes, experimental conditions, error representation); move numerical results, percentage changes, statistical conclusions, and interpretive commentary to the main text.

***Fig 1***

The figure title has been revised to remove “coupling” language (Revised MS, p. 15, Lines 290–295). Correlation coefficients and interpretive conclusions have been removed from panel captions; the key statistic (r = 0.25, p < 0.01, R² = 0.06) is retained as an in-panel label in Fig 1(D). Y-axis labels updated to Electrical Conductivity (μS/cm) throughout.

***Fig 2***

The caption no longer states ANOVA significance levels or includes phrases such as “consistent with leaching-driven downward redistribution” (Revised MS, p. 16, Lines 309–313). Moisture class definitions (empirical terciles within each depth interval) are noted instead.

***Fig 3***

Caption reduced from six lines to one line (Revised MS, p. 18, Lines 349–350):

*Fig 3. Seasonal heatmap of depth-resolved salinity dynamics (April–September 2025).*

The right-axis label has been changed from “Soil Salt Content (g/kg)” to “EC-derived Salinity Index (g/kg).”

***Fig 4***

The EC time series (green line, right-axis) has been removed, eliminating the visual redundancy of two near-identical trajectories. The caption has been simplified accordingly (Revised MS, p. 18, Lines 358–361).

***Fig 5***

The caption no longer includes the +329% surface surge, +42% subsurface increase, or specific salinity values; it describes what the panels show rather than what the numbers mean (Revised MS, p. 20, Lines 387–392). Subplot labels updated: (A) Soil Moisture, (B) EC-derived Salinity Index, (C) EC-derived Salinity Dynamics; Y-axis of (C) relabelled to EC-derived Salinity Index (g/kg).

***Fig 6***

The caption has been reduced to title and brief schematic description only (Revised MS, p. 22, Lines 436–437). All causal panel labels were softened or removed: “Downward Leaching” → “Downward Movement (inferred)”; “Insufficient Leaching” and “Critical window” deleted; accumulation and redistribution labels reworded to reflect observational rather than mechanistic interpretation. All r-values, p-values, and percentage annotations removed from the panel (see also Point 7-d).

***Fig 7***

The detailed decision-flow narrative was deleted. The caption was simplified to a single descriptive line (Revised MS, p. 25, Lines 489–490). Panel labels were updated to replace “Salt” with “Salinity” where appropriate, decision node time labels generalised to Early-, Mid-, and Late-Season, and the EC threshold in Decision Node 3 revised to ≤ 800 μS/cm. A caveat note was additionally inserted (Revised MS, p. 25, Lines 491–494) clarifying that the threshold values and management responses illustrated in the figure are intended as conceptual guidance based on observed patterns rather than prescriptive criteria, and should be adapted to specific local soil, crop, and hydrological conditions.

***Tables***

Table headers across Tables 1, 2, and 3 have been updated from “Salt Content (g/kg)” to “EC-derived Salinity (g/kg),” consistent with the global terminology revision described under Point 3.

We hope this revision demonstrates the care with which we have engaged with the reviewers’ concerns. The manuscript now presents a more rigorously worded and appropriately hedged account of our observational findings. We are happy to provide further clarification if needed.

Sincerely,

Xuan Chen

Corresponding Author

Department of Hydraulic and Civil Engineering, Hetao College

Bayannur, Inner Mongolia, China

E-mail: 412766722@qq.com
