# Supplementary material for: Depth-dependent soil water-salt relationships in the Hetao Irrigation District, arid Northwest China: Implications for sustainable management
Source: PLoS One. 2026 Oct 20;21(10):e0360976. doi: 10.1371/journal.pone.0360976 (PMC13682659; doi:10.1371/journal.pone.0360976)
Supplement: Peer review history

# Peer review: Decision Letter 0

**DOI:** 10.1371/journal.pone.0360976.r001

**Authors:** Debasis Mitra

24 Apr 2026

Dear Dr. Chen,

Thank you for submitting your manuscript to PLOS ONE. After careful consideration, we feel that it has merit but does not fully meet PLOS ONE’s publication criteria as it currently stands. Therefore, we invite you to submit a revised version of the manuscript that addresses the points raised during the review process.

Please submit your revised manuscript by Jun 07 2026 11:59PM. If you will need more time than this to complete your revisions, please reply to this message or contact the journal office at plosone@plos.org. When you're ready to submit your revision, log on to https://www.editorialmanager.com/pone/ and select the 'Submissions Needing Revision' folder to locate your manuscript file.

- A letter that responds to each point raised by the academic editor and reviewer(s). You should upload this letter as a separate file labeled 'Response to Reviewers'.
- A marked-up copy of your manuscript that highlights changes made to the original version. You should upload this as a separate file labeled 'Revised Manuscript with Track Changes'.
- An unmarked version of your revised paper without tracked changes. You should upload this as a separate file labeled 'Manuscript'.

If you would like to make changes to your financial disclosure, please include your updated statement in your cover letter. Guidelines for resubmitting your figure files are available below the reviewer comments at the end of this letter.

If applicable, we recommend that you deposit your laboratory protocols in protocols.io to enhance the reproducibility of your results. Protocols.io assigns your protocol its own identifier (DOI) so that it can be cited independently in the future. For instructions see: https://journals.plos.org/plosone/s/submission-guidelines#loc-laboratory-protocols. Additionally, PLOS ONE offers an option for publishing peer-reviewed Lab Protocol articles, which describe protocols hosted on protocols.io. Read more information on sharing protocols at https://plos.org/protocols?utm_medium=editorial-email&utm_source=authorletters&utm_campaign=protocols.

As the corresponding author, your ORCID iD is verified in the submission system and will appear in the published article. PLOS supports the use of ORCID, and we encourage all coauthors to register for an ORCID iD and use it as well. Please encourage your coauthors to verify their ORCID iD within the submission system before final acceptance, as unverified ORCID iDs will not appear in the published article. *Only*  the individual author can complete the verification step; PLOS staff *cannot*  verify ORCID iDs on behalf of authors.

We look forward to receiving your revised manuscript.

Kind regards,

Debasis Mitra

Academic Editor

PLOS One

Journal Requirements:

When submitting your revision, we need you to address these additional requirements.

1. Please ensure that your manuscript meets PLOS ONE's style requirements, including those for file naming. The PLOS ONE style templates can be found at

https://journals.plos.org/plosone/s/file?id=wjVg/PLOSOne_formatting_sample_main_body.pdf and

https://journals.plos.org/plosone/s/file?id=ba62/PLOSOne_formatting_sample_title_authors_affiliations.pdf

2. In your Methods section, please provide additional information regarding the permits you obtained for the work. Please ensure you have included the full name of the authority that approved the field site access and, if no permits were required, a brief statement explaining why.

3. If the reviewer comments include a recommendation to cite specific previously published works, please review and evaluate these publications to determine whether they are relevant and should be cited. There is no requirement to cite these works unless the editor has indicated otherwise.

[Note: HTML markup is below. Please do not edit.]

Reviewers' comments:

Reviewer's Responses to Questions

**Comments to the Author**

1. Is the manuscript technically sound, and do the data support the conclusions?

Reviewer #1: Yes

Reviewer #2: Yes

**********

2. Has the statistical analysis been performed appropriately and rigorously?

Reviewer #1: Yes

Reviewer #2: Yes

**********

3. Have the authors made all data underlying the findings in their manuscript fully available?

The PLOS Data policy

Reviewer #1: Yes

Reviewer #2: Yes

**********

4. Is the manuscript presented in an intelligible fashion and written in standard English?

Reviewer #1: Yes

Reviewer #2: Yes

**********

Reviewer #1: The manuscript addresses an important topic and contains valuable field data. However, substantial revisions are required before it can be considered for publication. The primary concerns relate to methodological limitations, overinterpretation of weak statistical relationships, formatting and language issues, and insufficient clarification of novelty. The following points are given below-

1. The manuscript claims to provide new insights into depth-dependent salinity dynamics. However, similar studies have already reported subsurface salt accumulation in arid irrigation systems. The authors should more clearly articulate what is novel in their work (e.g., scale of dataset, analytical framework, or methodological approach).

2. The reported correlation between soil moisture and salinity in deep layers (r = 0.25, R² = 0.06) is statistically significant but weak. The manuscript repeatedly interprets this relationship as evidence of meaningful coupling and redistribution processes. This interpretation appears overstated given the low explanatory power. The authors should clearly distinguish between statistical significance and practical significance

3. Salt content is calculated directly from electrical conductivity (EC), yet EC is subsequently used in regression analyses and to interpret salinity dynamics. This introduces an inherent circularity in the analytical framework. Although this limitation is acknowledged, it should be more explicitly discussed and accompanied by a more cautious interpretation of regression results and derived relationships.

4. The study is based on observations from a single growing season (2025), which limits its ability to support conclusions regarding long-term salinity dynamics or sustainability. Consequently, statements referring to “long-term accumulation” and “sustainability risks” are not fully substantiated by the dataset. The authors should revise these claims to reflect that the findings represent short-term observational evidence rather than long-term trends.

5. The absence of sampling in May, due to irrigation constraints, significant limitation, as this period likely coincides with peak leaching activity. The manuscript should more critically assess the implications of this data gap and avoid drawing strong conclusions about leaching dynamics in the absence of observations from this key period.

6. The stratified sampling approach deliberately includes sites across a range of salinity gradients to capture variability. While this design is appropriate for process-oriented analysis, it limits the representativeness of the results at the regional scale. The authors should clearly state that the findings are not intended for regional extrapolation and should be interpreted as indicative of patterns rather than representative averages.

7. The study relies exclusively on electrical conductivity (EC)-based estimates to infer soil salinity, without incorporating direct gravimetric measurements or ion-specific analyses (e.g., Na⁺, Cl⁻). This reliance constrains the accuracy of absolute salt quantification and prevents assessment of sodicity-related risks. This limitation should be more explicitly acknowledged and emphasized in both the Methods and Discussion sections.

8. Key findings, particularly those related to subsurface accumulation and weak correlations, are reiterated across multiple sections. The manuscript would benefit from improved conciseness by minimizing repetition.

9. All figures and tables should be clearly referenced and adequately described within the text. Captions should be revised to improve clarity and conciseness, avoiding unnecessary repetition of information already presented in the main text.

Reviewer #2: There is need to improve on language and use of scientific writing otherwise the manuscrip is good.

there are CV values that are too large >30% which are unacceptable in agriculture.

Discussion must not repeat results but show strong arguments

**********

what does this mean?). If published, this will include your full peer review and any attached files.

If you choose “no”, your identity will remain anonymous but your review may still be made public.

**Do you want your identity to be public for this peer review?**  For information about this choice, including consent withdrawal, please see our Privacy Policy

Reviewer #1: **Yes:**  Chandrashekhar Singh

Reviewer #2: No

**********

[NOTE: If reviewer comments were submitted as an attachment file, they will be attached to this email and accessible via the submission site. Please log into your account, locate the manuscript record, and check for the action link "View Attachments". If this link does not appear, there are no attachment files.]

To ensure your figures meet our technical requirements, please review our figure guidelines: https://journals.plos.org/plosone/s/figures

You may also use PLOS’s free figure tool, NAAS, to help you prepare publication quality figures: https://journals.plos.org/plosone/s/figures#loc-tools-for-figure-preparation.

NAAS will assess whether your figures meet our technical requirements by comparing each figure against our figure specifications.

Attachment Submitted filename: Reviewer,s Comments.docx

Attachment Submitted filename: PONE-D-26-08043 Reviewed.pdf

# Author comment: Author response to Decision Letter 1

**DOI:** 10.1371/journal.pone.0360976.r002

28 May 2026

Dear Editor and Reviewers,

Thank you very much for your constructive comments and valuable suggestions regarding our manuscript.

We have carefully addressed all reviewer comments and revised the manuscript accordingly. A detailed point-by-point response has been uploaded as a separate file entitled “Response to Reviewers” to preserve the formatting clarity and readability of the responses.

Please kindly refer to the attached document for our complete responses. We sincerely appreciate your time and thoughtful evaluation of our work.

Sincerely,

Xuan Chen

Attachment Submitted filename: Response_to_Reviewers.docx

# Peer review: Decision Letter 1

**DOI:** 10.1371/journal.pone.0360976.r003

**Authors:** Jianhong Zhou

4 Oct 2026

Depth-dependent soil water-salt relationships in the Hetao Irrigation District, arid Northwest China: Implications for sustainable management

PONE-D-26-08043R1

Dear Dr. Chen,

We’re pleased to inform you that your manuscript has been judged scientifically suitable for publication and will be formally accepted for publication once it meets all outstanding technical requirements.

Within one week, you’ll receive an e-mail detailing the required amendments. When these have been addressed, you’ll receive a formal acceptance letter and your manuscript will be scheduled for publication.

An invoice will be generated when your article is formally accepted. Please note, if your institution has a publishing partnership with PLOS and your article meets the relevant criteria, all or part of your publication costs will be covered. Please make sure your user information is up-to-date by logging into Editorial Manager at Editorial Manager® and clicking the ‘Update My Information' link at the top of the page. For questions related to billing, please contact billing support.

If your institution or institutions have a press office, please notify them about your upcoming paper to help maximize its impact. If they’ll be preparing press materials, please inform our press team as soon as possible -- no later than 48 hours after receiving the formal acceptance. Your manuscript will remain under strict press embargo until 2 pm Eastern Time on the date of publication. For more information, please contact onepress@plos.org.

Kind regards,

Jianhong Zhou

Staff Editor

PLOS One

Additional Editor Comments (optional):

Reviewers' comments:

Reviewer's Responses to Questions

**Comments to the Author**

Reviewer #1: All comments have been addressed

Reviewer #2: All comments have been addressed

**********

2. Is the manuscript technically sound, and do the data support the conclusions?

Reviewer #1: Yes

Reviewer #2: Yes

**********

3. Has the statistical analysis been performed appropriately and rigorously?

Reviewer #1: Yes

Reviewer #2: Yes

**********

4. Have the authors made all data underlying the findings in their manuscript fully available?

The PLOS Data policy

Reviewer #1: Yes

Reviewer #2: Yes

**********

5. Is the manuscript presented in an intelligible fashion and written in standard English?

Reviewer #1: Yes

Reviewer #2: Yes

**********

Reviewer #1: (No Response)

Reviewer #2: Authors addressed all raised comments. The authors just need to imporove language but this can be done bt typists

**********

what does this mean?). If published, this will include your full peer review and any attached files.

If you choose “no”, your identity will remain anonymous but your review may still be made public.

**Do you want your identity to be public for this peer review?**  For information about this choice, including consent withdrawal, please see our Privacy Policy

Reviewer #1: **Yes:**  Chandrashekhar Singh

Reviewer #2: **Yes:**  Andrew Tapiwa Kugedera

**********

Attachment Submitted filename: Reviewer,s Comments.docx

# Editor report: Acceptance letter

**DOI:** 10.1371/journal.pone.0360976.r004

**Authors:** Jianhong Zhou

PONE-D-26-08043R1

PLOS One

Dear Dr. Chen,

I'm pleased to inform you that your manuscript has been deemed suitable for publication in PLOS One. Congratulations! Your manuscript is now being handed over to our production team.

At this stage, our production department will prepare your paper for publication. This includes ensuring the following:

* All references, tables, and figures are properly cited

* All relevant supporting information is included in the manuscript submission,

* There are no issues that prevent the paper from being properly typeset

You will receive further instructions from the production team, including instructions on how to review your proof when it is ready. Please keep in mind that we are working through a large volume of accepted articles, so please give us a few days to review your paper and let you know the next and final steps.

Lastly, if your institution or institutions have a press office, please let them know about your upcoming paper now to help maximize its impact. If they'll be preparing press materials, please inform our press team within the next 48 hours. Your manuscript will remain under strict press embargo until 2 pm Eastern Time on the date of publication. For more information, please contact onepress@plos.org.

You will receive an invoice from PLOS for your publication fee after your manuscript has reached the completed accept phase. If you receive an email requesting payment before acceptance or for any other service, this may be a phishing scheme. Learn how to identify phishing emails and protect your accounts at https://explore.plos.org/phishing.

If we can help with anything else, please email us at customercare@plos.org.

Thank you for submitting your work to PLOS One and supporting open access.

Kind regards,

PLOS One Editorial Office Staff

on behalf of

Dr. Jianhong Zhou

Staff Editor

PLOS One
